# Molecular Characterization and Clinical Relevance of *MGMT*‐Silenced Pancreatic Cancer

**DOI:** 10.1002/cam4.70393

**Published:** 2024-12-02

**Authors:** Federico Nichetti, Marco Silvestri, Luca Agnelli, Andrea Franza, Chiara Pircher, Simone Rota, Paolo Ambrosini, Giuseppe Fotia, Jennifer Hüllein, Giovanni Randon, Panna Lajer, Federica Perrone, Elena Tamborini, Giuseppe Leoncini, Jorgelina Coppa, Michele Droz Dit Busset, Sara Pusceddu, Massimo Milione, Federica Morano, Filippo Pietrantonio, Giancarlo Pruneri, Vincenzo Mazzaferro, Daniel B. Lipka, Bruno Christian Köhler, Daniel Hübschmann, Stefan Fröhling, Filippo de Braud, Monica Niger

**Affiliations:** ^1^ Medical Oncology Department Fondazione IRCCS Istituto Nazionale dei Tumori di Milano Milan Italy; ^2^ Computational Oncology, Molecular Precision Oncology Program, National Center for Tumor Diseases (NCT) and German Cancer Research Center (DKFZ) Heidelberg Germany; ^3^ Department of Diagnostic Innovation, Second Division of Pathology Fondazione IRCCS Istituto Nazionale dei Tumori Milan Italy; ^4^ Division of Translational Medical Oncology, Section of Translational Cancer Epigenomics, German Cancer Research Center (DKFZ) and National Center for Tumor Diseases (NCT) Heidelberg Germany; ^5^ Department of Pathology and Laboratory Medicine, First Division of Pathology Fondazione IRCCS Istituto Nazionale dei Tumori Milan Italy; ^6^ Hepato‐Pancreato‐Biliary Surgery and Liver Transplantation Fondazione IRCCS Istituto Nazionale dei Tumori Milan Italy; ^7^ Department of Oncology and Hemato‐Oncology University of Milan Milan Italy; ^8^ Division of Translational Medical Oncology National Center for Tumor Diseases (NCT) Heidelberg, German Cancer Research Center (DKFZ) Heidelberg Germany; ^9^ Liver Cancer Center Heidelberg University Hospital Heidelberg Heidelberg Germany; ^10^ German Cancer Consortium (DKTK) Heidelberg Germany; ^11^ Pattern Recognition and Digital Medicine Group Heidelberg Institute for Stem Cell Technology and Experimental Medicine (HI‐STEM) Heidelberg Germany

**Keywords:** biomarker, KRAS wild type, MGMT, molecular profiling, pancreatic cancer, temozolomide

## Abstract

**Background:**

The identification of actionable molecular targets of pancreatic cancer (PAC) is key to improving patient outcomes. We hypothesized O6‐methylguanine‐DNA methyltransferase (*MGMT*) silencing may occur in a subset of PAC tumors, with unexplored clinical and molecular correlates.

**Experimental Design:**

We leveraged sequencing data from The Cancer Genome Atlas (TCGA), the Clinical Proteomic Tumor Analysis Consortium 3 (CPTAC‐3), and the (Australian Pancreatic Cancer Genome Initiative) PACA‐AU cohorts to characterize *MGMT*‐silenced PAC. Genomic, transcriptomic, methylation, and clinical data were investigated, and findings were validated in silico using methylation, transcriptomic and drug sensitivity data from Cancer Cell Line Encyclopedia (CCLE) project, and in a real‐world cohort of PAC patients profiled for MGMT status at Istituto Nazionale Tumori of Milan (INT).

**Results:**

On the basis of Human Methylation 450k data, *MGMT* silencing was identified in ~6% of PAC cases and was enriched in tumors with non‐ductal histology, with a trend toward longer overall survival. *MGMT*‐silenced tumors were associated with a lower frequency of *KRAS* mutations and showed features of immune exclusion. In the INT cohort, *MGMT*‐silencing was confirmed in ~7% of cases and prevalent in *KRAS* wild type tumors, with a favorable prognostic impact. In silico analysis suggested a higher sensitivity to alkylating and DNA damaging agents in *MGMT*‐silenced PAC cell lines.

**Conclusions:**

*MGMT* silencing occurs in a small subgroup of PACs and is enriched in *KRAS* wild type cases, with a favorable prognostic impact. Our findings provide the rationale to explore combinations of alkylating with DNA damaging agents in *MGMT*‐silenced PAC.

## Graphical abstract

O6‐methylguanine‐DNA methyltransferase (*MGMT*) silencing is a validated biomarker in glioblastoma but has not been deeply investigated in pancreatic cancer (PAC). We show that *MGMT* silencing occurs in a small but non‐negligible (~6% to 7%) subgroup of PAC patients, with a favorable prognostic impact. We also noted that *MGMT*‐silenced PAC is associated with a lower frequency of *KRAS* mutations and showed features of immune exclusion. In addition, in silico analysis suggests a higher sensitivity to alkylating and DNA damaging agents in *MGMT*‐silenced PAC cell lines. Our findings support a rationale to explore combinations of alkylating (including temozolomide) with DNA damaging agents in *MGMT*‐silenced PAC.

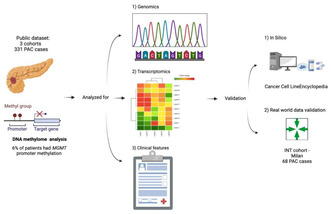

## 1 Introduction

Pancreatic cancer (PAC), mainly represented by pancreatic ductal adenocarcinoma, is a highly aggressive disease with an increasing incidence and a poor prognosis [1]. PAC is predicted to become the second leading cause of cancer‐related death by 2030, with still a 5‐year survival rate of < 10% [2]. In recent years, several studies have explored the molecular heterogeneity of PAC, revealing that up to 25% of these tumors harbor actionable alterations [3, 4, 5, 6, 7, 8, 9]. In patients harboring these alterations, molecularly matched therapies demonstrated a significant benefit compared to standard chemotherapy or untargeted treatments [10, 11]. However, despite these advances, most patients with PAC are treated with cytotoxic chemotherapy, with a disappointing overall survival (OS) of < 1 year. Therefore, the identification of clinically actionable molecular targets of PAC is key to improving patients' outcomes.

O^6^‐methylguanine‐DNA methyltransferase (*MGMT*) is a DNA repair enzyme that removes alkyl groups from the O^6^ position of guanine in DNA, and its activity is critical in the resistance of cancer cells to alkylating agents. *MGMT* promoter hypermethylation results in the suppression of its expression and to its inactivation, leading to an increased sensitivity of cancer cells to alkylating agents like temozolomide (TMZ) [12]. *MGMT* promoter hypermethylation is a validated biomarker for the efficacy of TMZ in glioblastoma [13], and is under active investigation in gastrointestinal malignancies, including colorectal cancers [14, 15, 16] and biliary tract cancers [17]. To date, the prevalence and impact of *MGMT* promoter hypermethylation in PAC are unknown.

The purpose of this study was to explore the frequency, the molecular correlates, and clinical significance of *MGMT* silencing in multi‐omics profiled cohorts of patients with PAC, and to explore the potential therapeutic implications of targeting *MGMT*‐silenced tumors.

## 2 Materials and Methods

The study flowchart is depicted in Figure S1.

Data of three PAC cohorts (the TCGA PAAD [The Cancer Genome Atlas pancreatic cancer, *n* = 178] [18], the CPTAC‐3 [Clinical Proteomic Tumor Analysis Consortium 3, *n* = 69] [19] and the PACA‐AU [Australian Pancreatic Cancer Genome Initiative, *n* = 84] [20]) with clinical, genomic, transcriptomic, and methylation profiles were explored and collected. *MGMT*‐silenced PAC cases were identified by analyzing CpG islands located in the *MGMT* promoter region. Next, the molecular landscape of *MGMT*‐silenced cases was explored and compared with *MGMT*‐active cases. An in silico validation of prior findings and a drug sensitivity analysis was performed on PAC cell lines with available transcriptomic, methylation, and drug sensitivity data. Finally, a real‐world cohort of PAC patients profiled for *MGMT* status was studied to validate previous findings.

### 2.1 Exploratory Cohort

*MGMT*‐silenced PAC cases were identified applying the *mgmtstp27* [21] algorithm on Illumina Human Methylation 450k raw data. Then, clinical, genomic, and transcriptomic profiles of *MGMT*‐methylated versus not‐methylated tumors were compared.

For RNAseq data, expression was used “as‐is,” that is, RNAseq data were not realigned and gene‐level expression estimates were downloaded and analyzed in terms of raw counts, fragments per kilobase per million reads (FPKM, for PACA‐AU), or transcripts per million (TPM, for TCGA and CPTAC‐3), harmonized against GRCh37. Batch‐corrected, variance‐stabilizing transformed (vst) count data were used to compare MGMT expression with its promoter methylation status.

Differential expression analysis was performed ed. with *DESeq2* [22] on count data, joint after batch correction by means of surrogate variable analysis [23] and the resulting list of most variable genes was used to perform a Gene Set Enrichment Analysis (GSEA) and to apply the PROGENy [24] algorithm, with the aim of identifying the most differentially active pathways in *MGMT*‐silenced PAC. Moreover, transcriptomic data were used also to infer (i) previously defined transcriptomic‐driven PAC subtypes (namely the Collisson, Moffitt, and Bailey subtypes [20, 25, 26]) and (b) the proportion of immune and stromal cells in the tumor microenvironment using deconvolution methods.

Genomic data, in terms of single nucleotide variants (SNVs), insertion‐deletions (indels), and copy number variations (CNVs, namely amplifications and deletions) were downloaded and compared “as‐is” according to *MGMT* status. Moreover, comparison of oncogenic pathway alteration frequencies among *MGMT*‐methylated and not‐methylated cases was performed, by considering that a case was altered in a given pathway if one or more pathway genes had a pathogenic alteration, as previously described [27]. Finally, differential representation of mutational signatures, both as COSMIC (Catalogue of Somatic Mutations in Cancer) and PCAWG (Pan‐Cancer Analysis of Whole Genomes) SNV signatures and as PCAWG Indel signatures, and of homologous recombination deficiency (HRD) between *MGMT*‐methylated and not‐methylated cases was assessed. A detailed description of bioinformatic methods for molecular data collection and analysis is provided in Data S1.

### 2.2 INT Validation Cohort

As a real‐world validation of previous findings, we investigated a cohort of consecutive PAC patients treated at Fondazione IRCCS Istituto Nazionale Tumori of Milan (INT). Starting 2017, all consecutive patients with a histologically confirmed diagnosis of PAC and with available archival tumor tissue that underwent successful MGMT status assessment were included. Baseline demographic, clinical and biological data were collected through an electronic database. All patients were followed up until death, loss to follow‐up, or data cutoff date (January 1, 2023).

Formalin‐fixed paraffin‐embedded (FFPE) samples from the most recent available tissue sample, either from the primary tumor or from a metastatic site, were used for molecular analyses. *MGMT* promoter methylation status was assessed by pyrosequencing (via the MGMT plus Diatech Pharmacogenetics [Jesi, Italy] kit), as previously described [17]. In detail, the assay reports the number of methylated CpG islands present in the promoter region of the *MGMT* gene, expressed as a percentage. The optimal threshold to define *MGMT* promoter methylation status was identified by means of the maximally selected rank statistic according to patients' overall survival (OS) [28]. Moreover, 4 micron‐thick slides were cut, deparaffinized, and reacted with monoclonal antibody against MGMT (Invitrogen, clone MT23.2, dilution 1:200). The immunohistochemical (IHC) assays were evaluated by an expert pathologist and nuclear immunolabeling was considered as positive, weakly positive, or negative based on the stain intensity, as compared to the internal control in normal cells, including endothelial cells and lymphocytes. Then, cases were defined as *MGMT*‐silenced or active according to the above dichotomization (above the defined percentage of methylated CpG islands) and the combined absence of MGMT expression at IHC.

Additional tumor molecular characterization was uniformly performed through the Ion Torrent Personal Genome platform (50 genes “Hotspot Cancer Panel, Ion Torrent”, Life Technologies Waltham, MA) as previously reported [17]. Clinical and molecular data were compared between *MGMT*‐silenced and active cases. The study was approved by the Institutional Review Board (INT 117/15) and written informed consent before tumor biopsy and/or request for molecular profiling was obtained from all alive patients at the time of study conduction.

### 2.3 In Silico Validation Cohort

Gene‐level normalized RNAseq (log2 transformed TPM values using a pseudo‐count of 1; version 22Q4) and methylation (as reported as methylation fraction of CpGs located 1 kilobase upstream the transcription start site [TSS], that is, not including cg12434587 and cg12981137) data of 52 PAC cell lines of the Cancer Cell Line Encyclopedia (CCLE) project were obtained through the Dependency Map (DepMap) portal of the Broad Institute [29]. Also, drug sensitivity data from the Genomics of Drug Sensitivity in Cancer (GDSC) project 2 dataset, as area under the dose–response curve (AUCs) values for screened PAC cell‐line/drug combinations, were downloaded from the DepMap portal. MGMT gene expression and methylation values were compared, to verify if methylation values could identify *MGMT*‐silenced cell lines. Moreover, for the same purpose, consensus clustering with K fixed at 2 using *cola* was performed on the expression matrix of genes previously identified as significantly differentially expressed (*p* < 0.005, absolute log2 fold change > 2.5) between *MGMT*‐methylated and not‐methylated human cases. The newly identified subgroups, inferred as *MGMT*‐methylated and not‐methylated, were then compared in terms of MGMT expression and sensitivity to anticancer compounds.

### 2.4 Statistical Analysis

The Fisher's exact test, chi‐squared test, and Wilcoxon–Mann–Whitney test were used to study the distribution of categorical and continuous variables, respectively, according to dichotomized *MGMT* status, as appropriate.

In the drug sensitivity analysis, the impact of MGMT expression on AUC values of different anticancer compounds was modeled as a dichotomous parameter in a univariate linear regression model. In detail, the optimal MGMT expression cutoff to distinguish the available cell lines according to the inferred‐*MGMT* methylation status was identified by means of ROC curve analysis, and the resulting groups were compared in terms of drug sensitivity. Given the limited number of cells, the filters for the selection of top candidate drugs were an adjusted *p* value cutoff of 0.2 and an absolute coefficient of the linear model of 1.4.

Median follow‐up was quantified with the reverse Kaplan–Meier estimator. Survival analysis methods were used to analyze overall survival (OS) in the public cohorts and in the INT cohort according to *MGMT* status. Survival curves and related descriptive statistics were obtained with the Kaplan–Meier method and comparisons between curves were performed with the logrank test. Univariable and multivariable Cox proportional models were designed using available clinical and biological annotations, and results were summarized using hazard ratios (HRs), together with the corresponding 95% confidence intervals (CI).

All *p* values were subjected to Benjamini–Hochberg multiple test correction when three or more tests were performed, and a two‐sided threshold of significance of 0.05 was set for all statistical evaluations unless otherwise specified. Statistical analyses were performed in R version 4.2.2 (2022‐10‐31) [R Foundation for Statistical Computing].

## 3 Results

### 3.1 *MGMT* Promoter Methylation Is a Rare Epigenetic Alteration in PAC

We assembled three publicly available PAC cohorts, for a total of 331 samples (Figure S1).

Among these, 50% were men and the majority (78%) had the primary tumor located in pancreatic head. Of note, this cohort comprised only 4% of patients with de novo metastatic disease.

Correlation analysis between MGMT RNAseq (vst) and M‐ and beta‐values of CpGs located on the *MGMT* promoter region confirmed that cg12434587 and cg12981137 had a significant, negative correlation with MGMT expression (Figure S2a).

The MGMT‐STP27 algorithm was run on preprocessed raw methylation data, separately for each individual study (TCGA, CPTAC‐3, and PACA‐AU). *MGMT* promoter methylation was identified in 11/179 (6.2%), 3/69 (4.3%), and 5/84 (5.9%) cases of the TCGA, CPTAC‐3, and PACA‐AU cohorts, respectively, for a total of 19/331 (5.7%) cases. As a further validation, *MGMT*‐methylated tumors had significantly lower MGMT expression compared to not‐methylated cases (Figure S2b).

Patients' and tumor characteristics according to *MGMT* methylation status are reported in Table 1.

**TABLE 1:** Patients' and tumor characteristics according to *MGMT* methylation status in the public cohorts.

Characteristics	Overall, *N* = 331	Methylated, *N* = 19	Not‐methylated, *N* = 312	*p*
Age	65 (58, 73)	64 (61, 71)	66 (58, 73)	0.8
Unknown	2	0	2	
Sex				
Female	166 (50%)	10 (53%)	156 (50%)	0.8
Male	164 (50%)	9 (47%)	155 (50%)	
Unknown	1	—	1	
Histology				
PDAC	307 (93%)	13 (68%)	294 (95%)	< 0.001
Rare subtype	23 (7.0%)	6 (32%)	17 (5.5%)	
Unknown	1	0	1	
Stage				
I	28 (8.7%)	2 (11%)	26 (8.6%)	0.7
II	268 (83%)	14 (78%)	254 (84%)	
III–IV	25 (7.8%)	2 (11%)	23 (7.6%)	
Unknown	10	1	9	
Moffit subtype				
Basal‐like	110 (33%)	4 (21%)	106 (34%)	0.2
Classical	221 (67%)	15 (79%)	206 (66%)	
Collisson subtype				
Classical	131 (40%)	7 (37%)	124 (40%)	0.7
Exocrine‐like	96 (29%)	7 (37%)	89 (29%)	
Quasimesenchymal	104 (31%)	5 (26%)	99 (32%)	
Bailey subtype				
ADEX	64 (19%)	7 (37%)	57 (18%)	0.026
Immunogenic	60 (18%)	—	60 (19%)	
Pancreatic progenitor	139 (42%)	10 (53%)	129 (41%)	
Squamous	68 (21%)	2 (11%)	66 (21%)	
Metabolic subtype				
Cholesterogenic	58 (18%)	3 (16%)	55 (18%)	0.6
Glycolytic	62 (19%)	3 (16%)	59 (19%)	
Mixed	50 (15%)	1 (5.3%)	49 (16%)	
Quiescent	161 (49%)	12 (63%)	149 (48%)	
Study				
CPTAC‐3	69 (21%)	3 (16%)	66 (21%)	> 0.9
PACA‐AU	84 (25%)	5 (26%)	79 (25%)	
TCGA	178 (54%)	11 (58%)	167 (54%)	

Abbreviations: ADEX, Aberrantly Differentiated Endocrine Exocrine; CPTAC‐3, Clinical Proteomic Tumor Analysis Consortium; PACA‐AU, Pancreatic Cancer Australia; PDAC, Pancreatic Ductal Adenocarcinoma; TCGA, The Cancer Genome Atlas Program.

Of note, *MGMT* was found methylated more frequently in samples with rare pancreatic cancer histology as compared to usual ductal adenocarcinomas.

### 3.2 Transcriptomic Landscape of *MGMT*‐Silenced PAC

**FIGURE 1:**
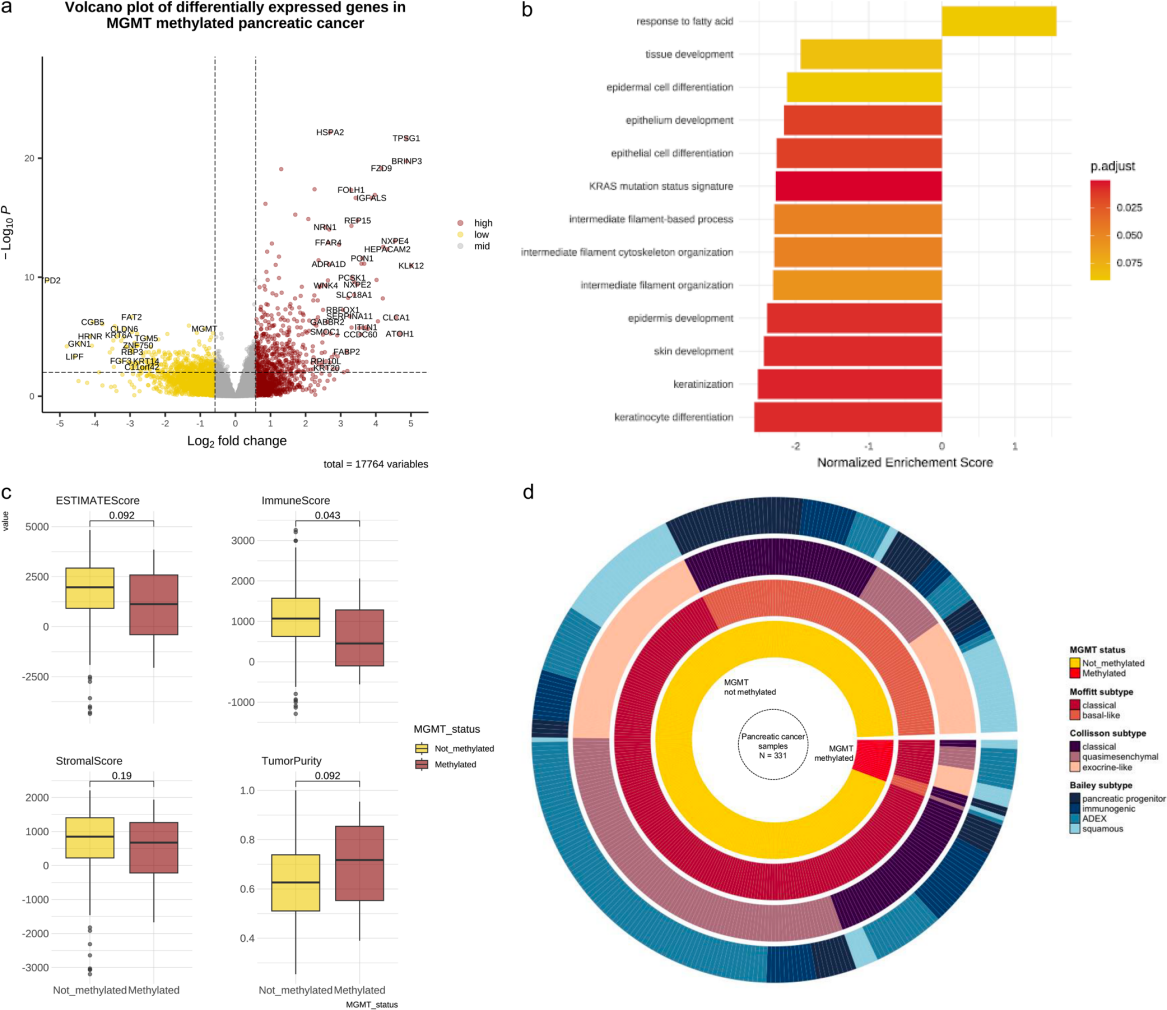
Transcriptomic features of *MGMT*‐silenced PAC. (a) Volcano plot of differentially expressed genes in *MGMT*‐methylated versus not‐methylated PAC. The vertical dotted lines identify genes with absolute log2FoldChange > 0.58 (i.e., absolute fold change > 1). The horizontal dotted line identifies genes with adjusted *p* < 0.01. Each point represents a gene, and genes are colored based on up (red) and downregulation (yellow) in *MGMT*‐methylated versus not‐methylated groups. (b) Bar plot reporting results of GSEA performed on up‐ and downregulated genes in *MGMT*‐methylated versus not‐methylated PAC. The color of the barplot reports the significance of the *p* value of the hypergeometric tests (after Benjamini–Hochberg multiple test correction). Only pathways with adjusted *p* < 0.1 are reported. Most pathways show a negative enrichment score, representing a negative enrichment in *MGMT*‐methylated tumors. (c) Boxplots comparing ESTIMATE deconvolution scores between *MGMT*‐methylated versus not‐methylated PAC. Wilcoxon mean rank‐sum *p* values are shown. (d) Overlay of PAC cases according to *MGMT*‐methylation status (inner ring) with published PAC transcriptomic subtypes (outer rings), according to Moffitt, Collisson, and Bailey classifications. ADEX, Aberrantly Differentiated Endocrine Exocrine; GSEA, Gene Set Enrichment Analysis; MGMT, O^6^ ‐methylguanine‐DNA methyltransferase; PAC, pancreatic cancer.

Next, we comprehensively characterized the impact of *MGMT* promoter methylation on tumor transcriptome. Differential expression analysis showed that 1881 and 1715 genes were significantly down‐ and upregulated, respectively, in *MGMT*‐methylated PACs (Figure 1a).

Conservative significance thresholds were applied to define a smaller list of genes for input to downstream pathway analysis. Among this list, GSEA revealed that MGMT‐methylated tumors had significantly lower enrichment in keratinocyte and epithelial cell differentiation pathways (Figure 1b), resembling the patterns previously described for KRAS‐wild type tumors compared to KRAS mutant [30]. Given this similarity, we tested the “KRAS mutation status signature,” as previously defined by Topham and colleagues [30]. MGMT‐methylated tumors showed a significant enrichment of the KRAS signature (*p* adjusted≤ 0.001); specifically, a negative enrichment of genes that resulted downregulated (*n* = 134) in KRAS mutants was highlighted (normalized enrichment score − 2.26, *p* adjusted < 0.001), suggesting a similarity between MGMT‐methylated and KRAS‐wild type tumors. Next, we used the binary cut method to summarize the list of negatively enriched pathways by clustering functional terms into groups; the similarity matrix (Figure S3) revealed that cell differentiation and immune response were among the most represented, negatively enriched pathways in MGMT‐methylated tumors.

Given this evidence, we applied in silico deconvolution methods to identify differential enrichment of immune cell populations. The ESTIMATE algorithm revealed that *MGMT*‐methylated tumors had significantly lower immune score; moreover, xCell analysis, cross‐checked with TIMER, confirmed a significant, negative enrichment of several immune cell types, suggesting that *MGMT* silencing may be associated with immune exclusion of PACs (Figure 1c; Figure S4).

Finally, to further understand the biological features of *MGMT* silencing, the activity of major signaling pathways was compared between *MGMT*‐methylated and not‐methylated cases. As shown in Figure S5, *MGMT*‐methylated tumors showed significantly lower enrichment scores for seven pathways, mostly for the JAK–STAT, NFkB, TNFalpha, TGFbeta, and EGFR pathways, while no pathway was significantly more active in this subgroup.

### 3.3 Expression‐Based Subtyping of *MGMT*‐Silenced PAC

Prior studies identified different methods to cluster PACs according to transcriptomic subtypes, with biological and clinical implications. The most widely adopted classification is the one proposed by Moffitt et al. [25], which categorizes tumors in classical and basal‐like subtypes. In our cohort (Figure 1d), *MGMT*‐methylated tumors showed an overrepresentation of classical subtype cases compared to not‐methylated cases (15/19, 79% vs. 206/312 66%, *p* = 0.2). Subtyping according to the Collisson [26] method revealed a nonsignificant numerical enrichment of exocrine‐like calls among *MGMT*‐methylated cases (7/19, 37% vs. 89/312, 29%, *p* = 0.7). Notably, we observed a significant difference in distribution of subtypes according to the Bailey [20] subtyping scheme (*p* = 0.026); in detail, in line with prior findings, *MGMT*‐methylated tumors had a significantly higher proportion of ADEX subtypes (7/19, 37% vs. 57/312, 18%), lower number of squamous cases (2/19, 11% vs. 66/312, 21%), and no cases of immunogenic tumors versus 60 (over 312, 19%). No significant difference was observed in distribution of metabolic subtypes distribution, according to the classification proposed by Karasinska et al. [30] (Figure S6).

### 3.4 Genomic Alterations of *MGMT*‐Silenced PAC

To characterize the somatic mutation landscape of *MGMT*‐methylated PAC, we performed an exploratory analysis in which the frequency of SNV/indels and copy number amplification/deletion events were compared between methylated and not‐methylated groups.

**FIGURE 2:**
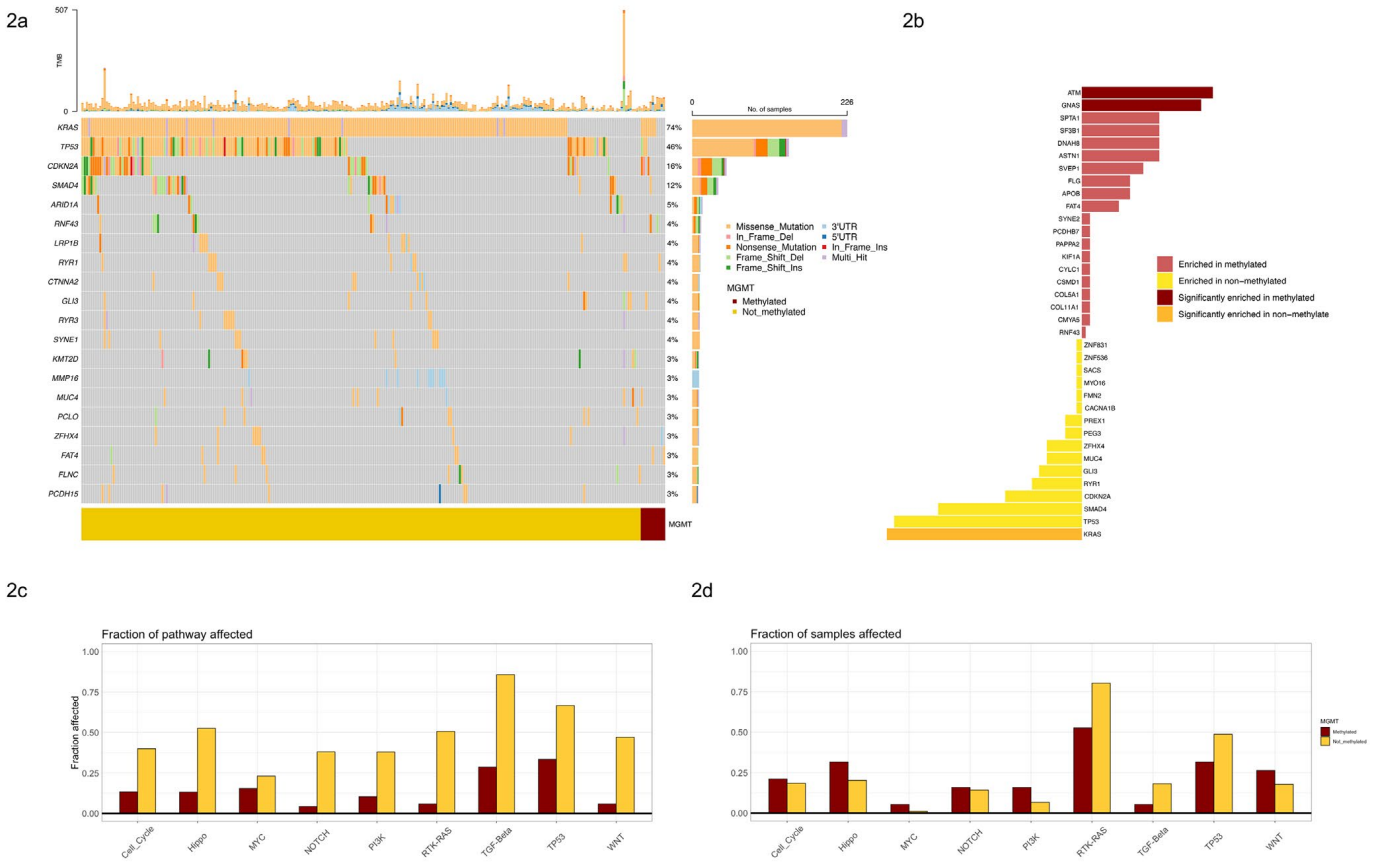
Genomic features of PAC cases according to *MGMT* methylation status. (a) Oncoprint summarizing somatic SNVs/indels according to *MGMT* methylation status. The top 20 most frequently mutated genes are presented. Upper bars represent TMB levels. *MGMT*‐methylated tumors are shown on the far right. (b) Bar plot reporting results of enrichment analysis of SNVs/indels according to *MGMT* methylation status. On the x axis, enrichment score is reported as the z‐score scaled‐, natural logarithm‐transformed odds ratio of the comparison between *MGMT*‐methylated versus not‐methylated cases. Bars are colored according to the enrichment score and adjusted (after Benjamini–Hochberg multiple test correction) *p* value as orange (significantly enriched in not‐methylated, enrichment score ≤ 0 and adjusted *p* ≤ 0.05), yellow (enriched in not‐methylated, enrichment score < 0 and adjusted *p* value > 0.05), light red (enriched in methylated, enrichment score > 0 and adjusted *p* > 0.05), and dark red (significantly enriched in methylated, enrichment score > 0 and adjusted *p* ≤ 0.05). (c, d) Barplots reporting results of oncogenic signaling pathways analysis according to *MGMT* methylation status. Fraction of each affected pathway (c) and fraction of samples affected (d). The *NRF2* pathway is not plotted as it was not affected in any cases. The * identifies the only significant different, as evaluated by Fisher's exact test. Del, Deletion—Ins, Insertion; MGMT, O^6^ ‐methylguanine‐DNA methyltransferase; NRF2, Nuclear Respiratory Factor 2; TMB, tumor mutational burden.

Overall, the median number of variants per sample was 32 and the top mutated genes were *KRAS*, *TP53*, *CDKN2A*, and *SMAD4*, as expected (Figure 2a). Of note, *BRCA1‐2* mutations were detected in six patients (*BRCA1 n* = 3; *BRCA2* *n* = 3), whereas *PALB2* mutation was detected in only one patient. When selecting for subgroup‐specific genes, we found that *KRAS* (36.8% vs. 76.0%, BH‐adjusted *p* = 0.020) and *TP53* (15.8% vs. 48.0%, BH‐adjusted *p* value = 0.069) mutations were significantly less frequent in *MGMT*‐methylated tumors, while *GNAS* (21.1% vs. 1.74%, BH‐adjusted *p* value = 0.020) and *ATM* (15.8% vs. 1.04%, BH‐adjusted *p* value = 0.043) mutations were significantly more prevalent in this subgroup (Figure 2b).

Moreover, for each subtype, 10 oncogenic signaling pathways [27] were used to compute the fraction of samples with at least one alteration in each pathway. This analysis confirmed that *MGMT*‐methylated tumors had a significantly lower frequency of mutations in the RTK‐RAS (*p* < 0.05) pathway (Figure 2c,d).

Supervised analysis of mutational signatures, both for SNVs and Indels, did not highlight any significant difference between *MGMT*‐methylated and not‐methylated cases (Figure S7a–d). Similarly, no significant prevalence of HRD cases was observed between the two subgroups.

Finally, in terms of somatic CNAs distribution, chromosome 6 (10%), 1 (8%), and 9 (7%) resulted to be the most altered genomic regions along the cohort while chromosome 21 the less frequently altered (1.5%). Interestingly, only the *MGMT*‐methylated group was characterized by specific CNAs events (*p* < 0.001) compared to the not‐methylated group, affecting genes like *KCNMB2* (42%), *ADO* (42%), and *ACAD11* (37%) (Figure S8).

### 3.5 Prognostic Impact of *MGMT* Silencing in PAC

**FIGURE 3:**
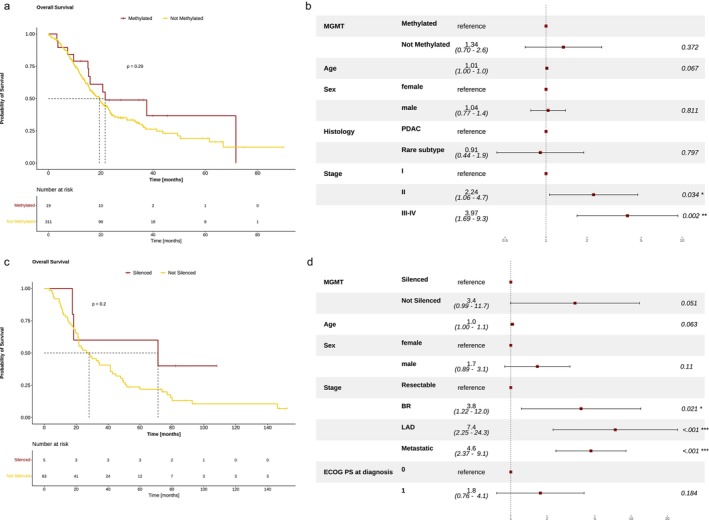
Prognostic impact of *MGMT*‐silencing in the public cohorts and in the INT validation cohort. (a) Overall survival represented through Kaplan–Meier curves according to *MGMT* methylation status in the pooled training cohort. Survival data are missing for one case among non‐methylated patients. (b) Forest plot of multivariable Cox regression analysis for overall survival adjusting for patients' age, sex, tumor histology, and tumor stage at diagnosis. (c) Overall survival represented through Kaplan–Meier curves according to *MGMT* methylation status in the INT cohort. (d) Forest plot of multivariable Cox regression analysis for overall survival adjusting for patients' age, sex, ECOG PS, and tumor stage at diagnosis. The hazard ratios are shown with 95% confidence intervals, and Wald *p* values are reported on the far right. Patient with *MGMT*‐methylated tumors show better overall survival after adjustment for other covariates. BR, Borderline Resectable; ECOG PS, Eastern Cooperative Oncology Group Performance Status; LAD, Locally Advanced Disease; MGMT, O^6^ ‐methylguanine‐DNA methyltransferase.

*MGMT*‐methylated PAC patients had a nonsignificant trend toward longer overall survival (OS, 21.7 vs. 19.5 months, HR 0.72 [95%CI 0.39–1.32], Wald test *p* = 0.26), which was confirmed using MGMT expression values (as a continuous variable) in a univariable and multivariable analysis (Figure 3a,b and Figure S9a,b).

### 3.6 Real‐World Validation of Clinical and Genomic Features of *MGMT* Silencing in PAC

From June 2017 to June 2021, 68 PAC patients with sufficient tumor tissue available for molecular profiling were included. Baseline characteristics are displayed in Table 2.

**TABLE 2:** Patients' and tumor characteristics according to *MGMT* status in the INT cohort.

Characteristics	Overall, *N* = 68	Silenced, *N* = 5	Not silenced, *N* = 63	*p*
Age	66 (57, 74)	59 (57, 72)	67 (58, 74)	0.6
Sex				
Female	27 (40%)	2 (40%)	25 (40%)	> 0.9
Male	41 (60%)	3 (60%)	38 (60%)	
Stage				
Resectable	31 (46%)	2 (40%)	29 (46%)	0.7
Borderline	5 (7.4%)	—	5 (7.9%)	
LAD	6 (8.8%)	1 (20%)	5 (7.9%)	
Metastatic	26 (38%)	2 (40%)	24 (38%)	
Surgery on primary tumor	41 (61%)	3 (60%)	38 (61%)	> 0.9
Unknown	1	—	1	
ECOG PS at diagnosis				
0	54 (82%)	3 (60%)	51 (84%)	0.2
1	12 (18%)	2 (40%)	10 (16%)	
Unknown	2	—	2	
*MGMT* promoter methylation	3.5 (2.0, 9.3)	14.0 (13.0, 14.0)	3.0 (2.0, 8.0)	0.003
MGMT expression by IHC				
Negative	16 (24%)	5 (100%)	11 (17%)	< 0.001
Positive	34 (50%)	—	34 (54%)	
Weakly positive	18 (26%)	—	18 (29%)	

Abbreviations: ECOG PS, Eastern Cooperative Oncology Group Performance Score; IHC, Immunohistochemistry; LAD, Locally Advanced Disease; MGMT, O^6^‐methylguanine‐DNA methyltransferase.

Of note, nearly 50% of patients had resectable or borderline resectable disease at diagnosis, and 60% underwent surgery for the primary tumor. Overall, 87% of cases were diagnosed with metastatic disease, either synchronous or metachronous. At data cutoff date, 57 (84%) patients had died, with a median follow‐up of 94.5 months (interquartile range [IQR]: 82.3–147.3) and a median OS of 28.1 months (IQR: 16.8–59.9). The best percentage cutoff of *MGMT* promoter methylation (as assessed by pyrosequencing) for OS prediction was identified as 10% (Figure S10), with 15 (22%) cases resulting as *MGMT*‐methylated and showing a trend toward longer OS (Figure S11a). No significant difference in OS was observed according to MGMT expression as evaluated by IHC (Figure S11b).

By combining pyrosequencing (with the 10% cutoff) and IHC (negative vs positive or weakly positive), we identified 5 (7.3%) cases as *MGMT*‐silenced. Clinical characteristics were similar between *MGMT*‐silenced and not silenced cases. As shown in the public cohorts, *MGMT*‐silenced cases had a trend toward longer OS (71.3 vs. 28.1 months, *p* = 0.208, Figure 3c), which resulted significant in a multivariable analysis including patients' and tumor characteristics (Figure 3d). Finally, we explored the mutational landscape, as evaluated by targeted genomic profiling, according to *MGMT* status: interestingly, we confirmed that *MGMT*‐silenced cases had a lower frequency of *KRAS* mutations (2/5, 40% vs. 57/63, 90%, *p* = 0.014, Figure S12).

### 3.7 In Silico Analysis of *MGMT*‐Silenced PAC Cell Models and Drug Sensitivity

Next, we explored whether *MGMT*‐methylated tumors would show distinct sensitivity to various anticancer compounds. We first analyzed gene expression and DNA methylation data from 52 human PAC cell lines from the Cancer Cell Line Encyclopedia. The lack of cg12434587 and cg12981137 among evaluated methylation sites resulted in no significant correlation between *MGMT* methylation values and expression, as expected (Figure S13). Therefore, to identify cell lines that would molecularly mimic *MGMT* promoter methylated PAC, we evaluated the expression pattern of genes that were found as strongly (adjusted *p* < 0.005 and log2FC > 2.5) up‐ (*n* = 56) and downregulated (*n* = 55) in *MGMT*‐methylated cases of the human cohorts. Consensus clustering performed on the expression matrix of this 111 gene set identified 5/52 (9.6%) cell lines that could be considered as representative of *MGMT*‐methylated PAC, with MGMT expression confirmed as significantly lower in these five cells (Figure S14a–c).

Anticancer drug screen data were then used to assess differential drug sensitivity according to *MGMT* status. Only a limited number of cells (*n* = 27/52, 51.9%) was screened, among which only one cell line was here classified as *MGMT*‐methylated, thus preventing a meaningful comparison between these two groups. Therefore, regression models to predict each drug's AUC were evaluated according to MGMT expression, here dichotomized according to the optimal cutoff to distinguish inferred‐*MGMT*‐methylated from *MGMT*‐not methylated cell lines (Figure S15).

As a result, 7 (25.9%) MGMT‐high and 20 (74.1%) MGMT‐low expression cell lines were compared. High MGMT expression was associated with significantly higher AUC (i.e., lower sensitivity) of anticancer compounds targeting DNA repair defects, including the PARP inhibitor Niraparib and the ATR inhibitor AZD6738, together inhibitors of the EGFR signaling (i.e., Erlotinib) and chromatin remodeling pathways. As expected, higher sensitivity to alkylating agents like temozolomide is observed with low MGMT expression (Table S1; Figure S16).

## 4 Discussion

We hypothesized that, similarly to other GI malignancies, *MGMT* inactivation due to gene promoter hypermethylation may occur in a subset of PAC patients, with specific molecular correlates and clinical implications. Here, we showed that *MGMT* promoter methylation occurs in ~6% of PAC cases. Despite different cutoffs adopted, this prevalence is lower as compared to other GI tumors like colorectal cancers where *MGMT* promoter methylation has been described in up to 40% of cases. Moreover, *MGMT* methylation was more frequently observed in non‐ductal, rare PAC subtypes, and in *KRAS*‐wild type tumors, thus defining it as a rare molecular alteration in this disease.

As shown for colorectal cancer [12], a qualitative definition of *MGMT* status (methylated vs. not methylated) may not fully portrait the biological activity of this gene; indeed, despite a significant difference in terms of MGMT expression, not all cases identified as *MGMT*‐methylated in our cohort had low‐to‐absent gene expression. This classification issue could impact the predictive role of *MGMT* for treatment with alkylating agents; in fact, previous studies have shown an optimal negative predictive value only for IHC, which can be considered a surrogate of gene expression that can be used in the clinical context [31]. Similarly, in the INT validation cohort, 22% of patients resulted *MGMT*‐methylated by pyrosequencing, but only five patients had combined *MGMT* methylation and lack of MGMT expression by IHC. Therefore, the proportion of *MGMT‐*silenced cases in the INT cohort was comparable to that identified in the analysis of the public datasets, with similar clinical and molecular features.

This prevalence of *MGMT*‐silencing is not negligible in the context of pancreatic cancer, where the only validated actionable biomarkers so far are germline *BRCA1‐2/PALB2* (for platinum salts and PARP inhibitors) [4] and *KRAS G12C* (for direct inhibitors [32]) mutations, which are found in ~5% and ~1% of cases, respectively.

As a major finding, in our study, *MGMT*‐silencing was associated with transcriptomic and genomic features of *KRAS* wild‐type PAC [30]. In detail, in both cohorts, we observed a significantly lower prevalence of *KRAS* mutations in *MGMT*‐methylated cases. This evidence is counterintuitive as compared to prior studies in colorectal cancer, where *MGMT* methylation was associated with an increase of G‐to‐A *KRAS* point mutations. In this light, PAC represents a different scenario that is poorly comparable to other tumors, as *KRAS* is known to be mutated in ~90% cases, representing the main genomic driver in most tumors and unlikely to be *MGMT*‐driven. However, this association with *KRAS* status is significant, as (a) it may help to refine the selection of cases to test for *MGMT* in future validation studies, (b) it may justify the survival advantage (although limited) that we observed in *MGMT*‐methylated cases; in this light, prior observational studies are in line with our findings, showing that MGMT‐silenced cases (with MGMT evaluated by IHC) had longer survival compared with MGMT expressing cases [33, 34]; and finally, (c) *MGMT* methylation could add to the plethora of molecular targets that are known to be enriched in *KRAS* wild‐type PAC.

As an additional finding, *MGMT*‐silenced cases were enriched in *GNAS* mutations (see Figure 2B). *GNAS* mutations have been previously associated with cystic pancreatic neoplasms, especially intraductal papillary mucinous neoplasms (IPMN), acting as cancer initiating drivers and being conserved between precursor lesions and transformed malignant tumors [35]. Of note, *GNAS* mutations in IPMNs have also been associated with favorable prognosis [36], aligning to our evidence. Together with the previously described genome hypermethylation and potential *GNAS* mutation‐related upregulation of lipid metabolism (as shown in Figure 1B), this evidence suggests that *MGMT* silencing being a feature of IPMN‐derived PDAC. Future studies are needed to evaluate this hypothesis.

As for other tumor types, prior preclinical evidence showed that *MGMT*‐silencing, spontaneously or via O(6)‐benzyl guanine, results in PAC growth inhibition and increased sensitivity to gemcitabine and to alkylating agents, including temozolomide [37, 38, 39]. Despite the limited amount of available data, we were able to confirm this rationale in silico, suggesting the potential utility of combining TMZ with DNA‐damaging agents in *MGMT*‐silenced cases. In this light, we also observed an enrichment of *ATM* gene mutations in *MGMT*‐methylated cases. *ATM* mutations have been associated with an HRD phenotype in different tumors, although their impact specifically in PDAC is still debated [40]. Also, clinical experience with temozolomide in PAC has been limited. A pivotal phase II trial tested TMZ alone in 15 unselected and not previously treated PAC patients, showing disease progression as best response in all cases [41]. These data highlight the need for careful patient selection and of combination strategies in case of future TMZ‐based trials in this hard‐to‐treat population.

Finally, even if proof of concept in its nature, here GSEA and deconvolution analysis suggests that *MGMT*‐methylated tumors may be associated with an immune‐excluded profile, as confirmed by the absence of Bailey's immunogenic cases in this subgroup. These data warrant further investigation, as prior studies showed that TMZ treatment may result in an immunogenic transformation of MGMT‐silenced colorectal tumors and thus sensitivity to immune‐checkpoint inhibitors [14, 42].

Our study has limitations in its design and sources. Our primary analysis is performed on three publicly available cohorts, which are therefore heterogeneous and mainly represented by not‐metastatic PAC cases that underwent surgery. As a consequence, these cohorts may not fully recapitulate real‐life PAC cases; for example, we observed a low overall rate of *KRAS* (~74%) and of *BRCA1‐2/PALB2* (~2%) mutations as compared to prior studies. As a result, while HRD is known to be reported in up to 25% of PAC cases [43], we could not observe a relevant proportion of cases with HRD genomic features. Furthermore, gene fusions/rearrangements data were not available in a uniform format for a joint analysis, so that this layer was not explored. Similarly, the INT validation cohort is relatively small sized and retrospective in nature, including both patients who did and did not develop metastatic disease, and *MGMT* status was here assessed with a different method. Nonetheless, we were able to validate our findings, thus adding robustness to our results.

In conclusion, here we described for the first time the prevalence and the clinical and molecular features of *MGMT*‐silenced PAC. Our findings provide the rationale to prospectively explore the efficacy of alkylating agents like TMZ in this rare population in clinical trials, possibly in combinations with DNA damaging agents or with active chemotherapy regimens in PAC.

## Author Contributions

**Federico Nichetti:** conceptualization (lead), data curation (lead), formal analysis (lead), funding acquisition (lead), investigation (equal), methodology (lead), project administration (equal), resources (equal), software (lead), supervision (lead), validation (equal), visualization (lead), writing – original draft (lead), writing – review and editing (lead). **Marco Silvestri:** conceptualization (equal), data curation (lead), formal analysis (lead), funding acquisition (equal), investigation (equal), methodology (equal), project administration (equal), resources (supporting), software (lead), supervision (lead), validation (lead), visualization (equal), writing – original draft (lead), writing – review and editing (lead). **Luca Agnelli:** software (equal), supervision (equal). **Andrea Franza:** software (equal), supervision (equal). **Chiara Pircher:** software (equal), validation (equal). **Simone Rota:** data curation (equal), visualization (equal). **Paolo Ambrosini:** data curation (equal). **Giuseppe Fotia:** investigation (equal). **Jennifer Hüllein:** formal analysis (equal). **Giovanni Randon:** validation (equal). **Panna Lajer:** conceptualization (equal). **Federica Perrone:** supervision (equal). **Elena Tamborini:** project administration (equal). **Giuseppe Leoncini:** formal analysis (equal), investigation (equal). **Jorgelina Coppa:** methodology (equal). **Michele Droz Dit Busset:** validation (equal). **Sara Pusceddu:** visualization (equal). **Massimo Milione:** resources (equal). **Federica Morano:** conceptualization (equal). **Filippo Pietrantonio:** supervision (equal). **Giancarlo Pruneri:** supervision (equal). **Vincenzo Mazzaferro:** supervision (equal). **Daniel B. Lipka:** methodology (equal). **Bruno Christian Köhler:** investigation (equal), methodology (equal), resources (equal), supervision (equal). **Daniel Hübschmann:** methodology (equal), resources (equal), supervision (equal). **Stefan Fröhling:** methodology (equal), supervision (lead). **Filippo de Braud:** supervision (lead). **Monica Niger:** conceptualization (equal), investigation (lead), project administration (lead), resources (equal), supervision (lead), visualization (equal).

## Conflicts of Interest

SP: received honoraria from Novartis, Ipsen, Pfizer, MerckSerono, and Advanced Accelerator Applications–AAA; received institutional research grant by Ipsen, Pfizer. FP: received honoraria from Servier, Bayer, Takeda, Merck‐Serono, Amgen, Pierre‐Fabre, MSD, BMS, Astellas, and GSK; research grants from Incyte, BMS, Astrazeneca, Agenus, and Amgen. FdB: Consulting: BMS, Pierre Fabre, Mattioli 1885, MCCann Health, MSD, IQVIA, Novartis; Advisory arrangements: Tiziana Life Sciences, BMS, Celgene, Novartis, Servier, Pharm Research Associated, Daiichi Sankyo, Ignyta, Amgen, Pfizer, Octimet Oncology, Incyte, Pierre Fabre, Eli Lilly, Roche, AstraZeneca, Gentili, Dephaforum, Merck & Co., Kenilworth, NJ, Bayer, Fondazione Menarini, Sanofi, Incyte, Taiho; Speaker's fees from BMS, Roche, Merck & Co., Kenilworth, NJ, Bayer, Ignyta, Dephaforum, Biotechespert, Prime Oncology, Pfizer, Nadirex, Ambrosetti, Incyte, Motore Sanità, Events, Fare Comunicazione, Itanet, Nadirex, ESO, AccMed, Idea‐z; Grants/contracts: research, unrestricted, and/or travel: Novartis, Roche, BMS, Celgene, Incyte, NMS, the healthcare business of Merck KGaA, Darmstadt, Germany, Kymab, Pfizer, Tesaro, and Merck & Co., Kenilworth, NJ; Traveling expenses from Bristol Myers Squibb, Roche, Celgene, and Amgen. MN: received Travel expenses for meetings from Astrazeneca, speaker honorarium from Accademia della Medicina and Incyte; honoraria from Sandoz, Medpoint SRL and Servier for editorial collaboration. Consultant honoraria from EMD Serono, Basilea Pharmaceutica, Servier, Incyte, MSD Italia, AstraZeneca, and Taiho. S.F.: Consulting or advisory board membership: Bayer, Illumina, Roche; honoraria: Amgen, Eli Lilly, PharmaMar, Roche; research funding: AstraZeneca, Pfizer, PharmaMar, Roche; travel or accommodation expenses: Amgen, Eli Lilly, Illumina, PharmaMar, and Roche.

## Supporting information


**Figure S1.** MGMT methylation in PAC analysis methods pipeline. Genome methylation data from three cohorts (TCGA, CPTAC‐3, and PACA‐AU, for a total of 331 cases) were analyzed to identify MGMT‐methylated cases. Clinical, genomic, and transcriptomic data were then explored and compared according to this new classification. Then, a cohort of 69 PAC patients treated and profiled for MGMT status with pyrosequencing and IHC and with targeted panel NGS at Fondazione IRCCS Istituto Nazionale dei Tumori was explored to validate previous findings; in this cohort, clinical and genomic data according to MGMT status were compared. CPTAC‐3, Clinical Proteomic Tumor Analysis Consortium—cohort 3; IHC, Immunohistochemistry; MGMT, O6‐methylguanine‐DNA methyltransferase; NGS, Next Generation Sequencing; PAC, Pancreatic Cancer; PACA‐AU, Pancreatic Cancer Australia; TCGA, The Cancer Genome Atlas Program.
**Figure S2.** (a) Correlogram showing correlation between MGMT expression values (vst) and beta‐ and M‐values of CpG islands located in the MGMT promoter region. While most CpGs had a weak, negative correlation with MGMT expression, the cg12434587 and cg12981137, included in MGMT‐STP27 had a significant, negative correlation with MGMT expression. The size of each ellipsis in the plot corresponds to the strength of the correlation. (b) Boxplots comparing MGMT expression (vst values) according to MGMT‐methylation status as defined by the MGMT‐STP27 algorithm. Density plots located on the right of the boxplots report the frequency of cases for each expression value. Wilcoxon mean rank‐sum *p* values are shown.MGMT, O6‐methylguanine‐DNA methyltransferase.
**Figure S3.** Heatmap with word cloud annotation of clustered Gene Ontology terms from GSEA of differentially expressed genes between MGMT‐methylated versus not‐methylated PAC cases. Enrichment is done on keywords compared to Gene Ontology background vocabulary and the significance corresponds to the font size of the keywords.GO Terms, Gene Ontology Terms; GSEA, Gene Set Enrichment Analysis; MGMT, O6‐methylguanine‐DNA methyltransferase.
**Figure S4.** Bar plot reporting results of linear regression analysis testing differences in cell subtypes composition in MGMT‐methylated versus not‐methylated PAC. Cell enrichment scores were obtained via xCell deconvolution performed on up‐ and downregulated ranked genes between MGMT‐methylated versus not‐methylated PAC cases. ****, ***, **, and * indicate adjusted (after Benjamini–Hochberg multiple test correction) *p* values < 1e‐6, 0.01, 0.05, and 0.1, respectively. cDC, conventional dendritic cells; CMP, Common myeloid progenitors; DC, Dendritic cells; iDC, immature dendritic cells; MGMT, O6‐methylguanine‐DNA methyltransferase; PAC, Pancreatic Cancer.
**Figure S5.** Bar plot reporting results of linear regression analysis testing differences in pathway activity analysis in MGMT‐methylated versus not‐methylated PAC. Pathway enrichment scores were calculated via the PROGENy algorithm performed on up‐ and downregulated ranked genes between MGMT‐methylated versus not‐methylated PAC cases. * indicate adjusted (after Benjamini–Hochberg multiple test correction) *p* < 0.05. MGMT, O6‐methylguanine‐DNA methyltransferase; PAC, Pancreatic Cancer; PROGENy, Pathway RespOnsive GENes for activity inference.
**Figure S6.** Scatterplot showing median expression levels of coexpressed glycolytic (x‐axis) and cholesterogenic (y‐axis) genes in each PAC sample. Metabolic subgroups were assigned on the basis of the relative expression levels of glycolytic and cholesterogenic genes. PAC, Pancreatic cancer.
**Figure S7.** Mutational signatures analysis. (a, b) Absolute exposures of SNVs (a) and indels (b) mutational signatures according to MGMT methylation status. (c, d) Heatmap representing hierarchical clustering of samples and signatures based on the relative exposures of the signatures. MGMT, O6‐methylguanine‐DNA methyltransferase; SNVs, Single nucleotide variants.
**Figure S8.** Barplots reporting enriched gene CNVs in MGMT‐methylated PAC. Each bar reports the proportion of cases with a CNV on a specific gene in MGMT‐methylated (upper half) and in not‐methylated (lower half) cases. Only significant genes are reported, according to Fisher’s exact test.CNVs, Copy number variants; MGMT, O6‐methylguanine‐DNA methyltransferase.
**Figure S9.** Prognostic impact of MGMT expression in the pooled training cohort. (a) Overall survival represented through Kaplan–Meier curves according to MGMT expression (vst). (b) Forest plot of multivariable Cox regression analysis for overall survival adjusting for patients’ age, sex, tumor histology, and tumor stage at diagnosis. The hazard ratios are shown with 95% confidence intervals, and Wald *p* values are reported on the far right. MGMT, O6‐methylguanine‐DNA methyltransferase; PAC, Pancreatic cancer.
**Figure S10.** Standardized log rank statistic according to MGMT promoter methylation values distribution. The maximally selected rank statistic method is used to identify the best cut point to dichotomize patients as MGMT‐Methylated or Mot‐Methylated. MGMT, O6‐methylguanine‐DNA methyltransferase; percent_met, percentage of methylated CpG islands present in the promoter region of the MGMT.
**Figure S11.** Exploratory analysis of prognostic impact of MGMT‐silencing in the INT validation cohort. (a) Overall survival represented through Kaplan–Meier curves according to MGMT promoter methylation value (as evaluated via pyrosequencing), dichotomized according to the maximally selected rank statistic. (b) Overall survival represented through Kaplan–Meier curves according to MGMT status assessed by IHC reported on three levels. IHC, Immunohistochemistry; MGMT, O6‐methylguanine‐DNA methyltransferase percent_met; neg, negative; percent_met, percentage of methylated CpG islands present in the promoter region of the MGMT gene; pos, positive.
**Figure S12.** Oncoprint summarizing somatic SNVs/indels according to MGMT status in the INT cohort. The top 15 most frequently mutated genes are presented. MGMT‐silenced tumors are shown on the far right. SNVs, Single nucleotide variants; MGMT, O6‐methylguanine‐DNA methyltransferase.
**Figure S13.** Scatterplot reporting the correlation between MGMT expression (log2(tpm + 1 values)) and TSS methylation value. The gray line refers to Pearson’s correlation coefficient with *p* value, also reported on the upper right quadrant of the plot. Dots are colored according to inferred MGMT‐methylation status. MGMT, O6‐methylguanine‐DNA methyltransferase; TSS, Transcription start site.
**Figure S14.** Identification of cell lines that transcriptionally mimic MGMT‐methylated PACs. (a) Heatmap depicting consensus clustering solution with k = 2. (b) Heatmap for the signature rows for k = 2. Cases are clustered using the Coefficient of variance and hierarchical clustering method. Gene expression is reported as z‐score values. (c) Boxplots comparing MGMT expression [log2(tpm + 1 values)] value according to inferred MGMT‐methylated PAC cell lines. Density plots located on the right of the boxplots report the frequency of cases for each expression value. Wilcoxon mean rank‐sum *p* value is shown. MGMT, O6‐methylguanine‐DNA methyltransferase; PAC, pancreatic cancer; Prob, probability; rel_diff, relative difference.
**Figure S15.** ROC curve identifying the optimal cutoff of MGMT expression value [log2(tpm + 1 values)] to distinguish cell lines that transcriptionally mimic MGMT‐methylated versus MGMT‐not methylated PACs. The numeric cutoff value is reported within the plot. MGMT, O6‐methylguanine‐DNA methyltransferase; PAC, Pancreatic cancer; ROC, Receiver operating characteristic.
**Figure S16.** Drug sensitivity according to MGMT expression. The volcano plot reports T statistic values of the linear regression analysis testing the impact of MGMT expression [log2(tpm + 1 values), dichotomized according to the optimal cutoff to distinguish inferred‐*MGMT* status as in Figure S15] on AUC values of anticancer compounds tested within the GDSC project. Higher T statistic values correspond to drugs that have higher AUC (i.e., lower sensitivity) with high MGMT expression values. Drugs are colored according to the T statistic and adjusted (after Benjamini–Hochberg multiple test correction) *p* value as red (T statistic > 1.4 and adjusted *p* value ≤ 0.20), yellow (T statistic > 1.4 and adjusted *p* ≤ 0.20), or gray (adjusted *p* > 0.20). AUC, Area under the curve; GDSC, Genomics of Drug Sensitivity in Cancer; MGMT, O^6^ ‐methylguanine‐DNA methyltransferase.

Table S1.

Data S1.

## Data Availability

Code is available upon reasonable request to the corresponding author. Data of the TCGA PAAD, CPTAC‐3, and PACA‐AU cohorts are publicly available, as detailed in Supplementary Methods. Data of the INT cohort are available upon reasonable request to the corresponding author.
