# Supplementary material for: Molecular Characterization and Clinical Relevance of MGMT‐Silenced Pancreatic Cancer
Source: Cancer Med. 2024 Dec 2;13(23):e70393. doi: 10.1002/cam4.70393 (PMC11609587; doi:10.1002/cam4.70393)

Supplementary Fig 1

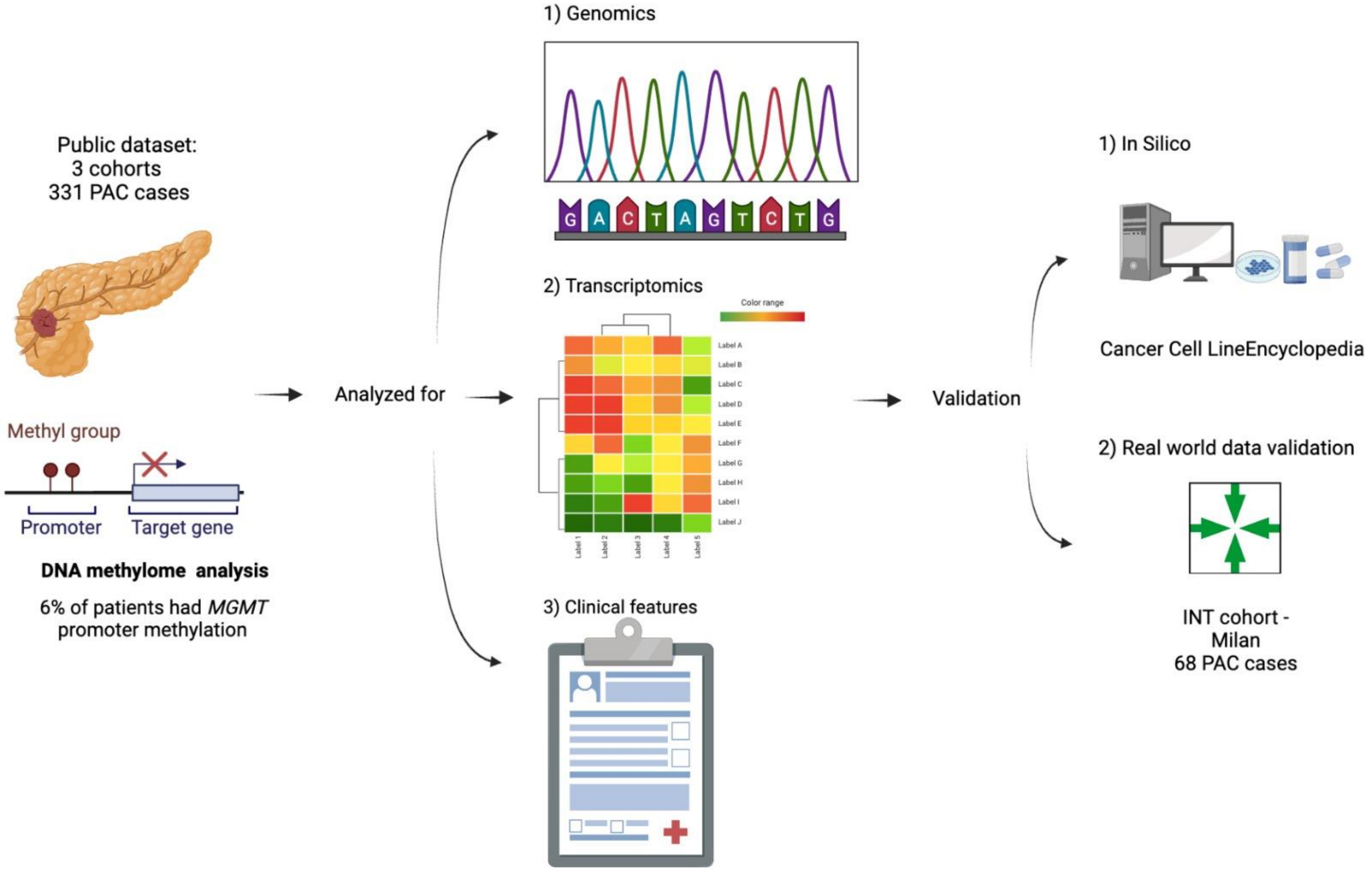

Supplementary Fig 2a-b

s2a

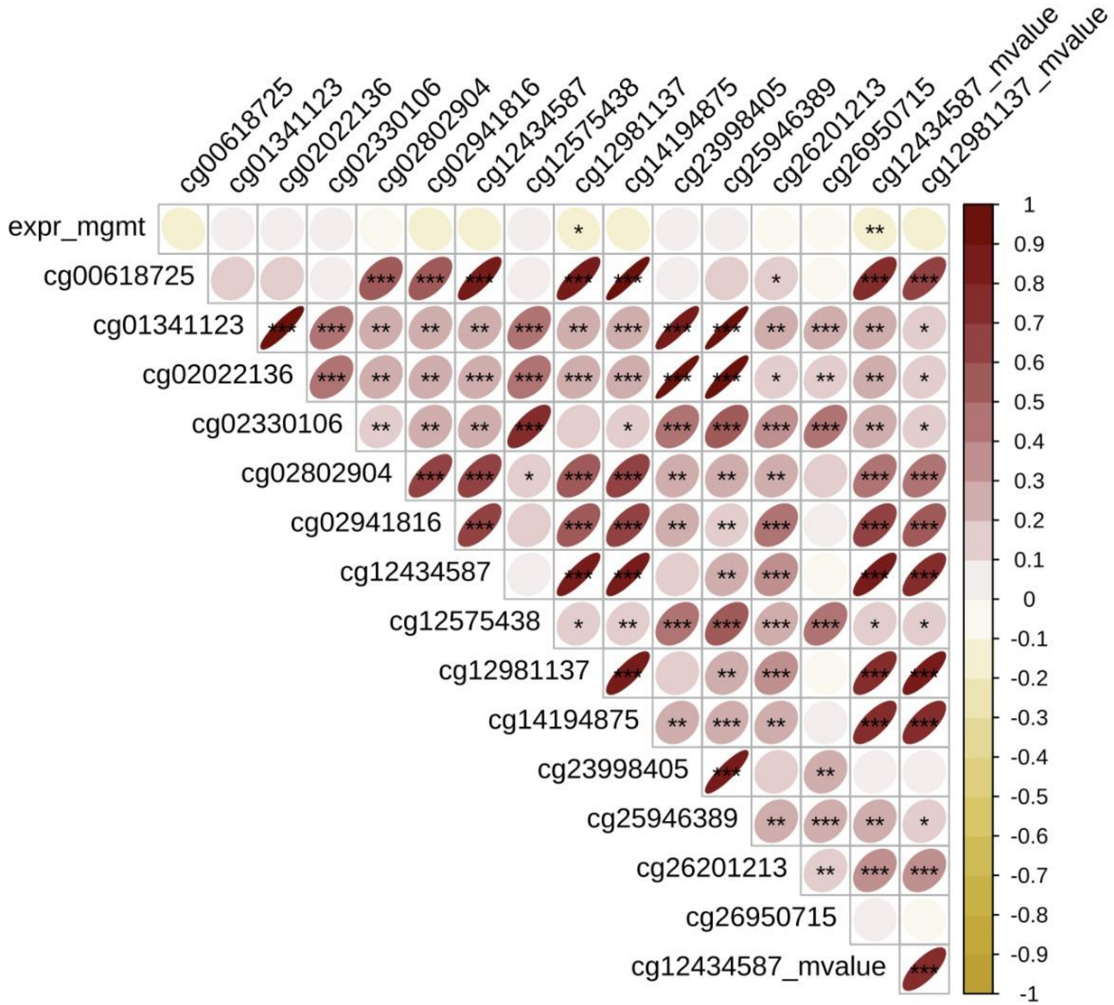

s2b

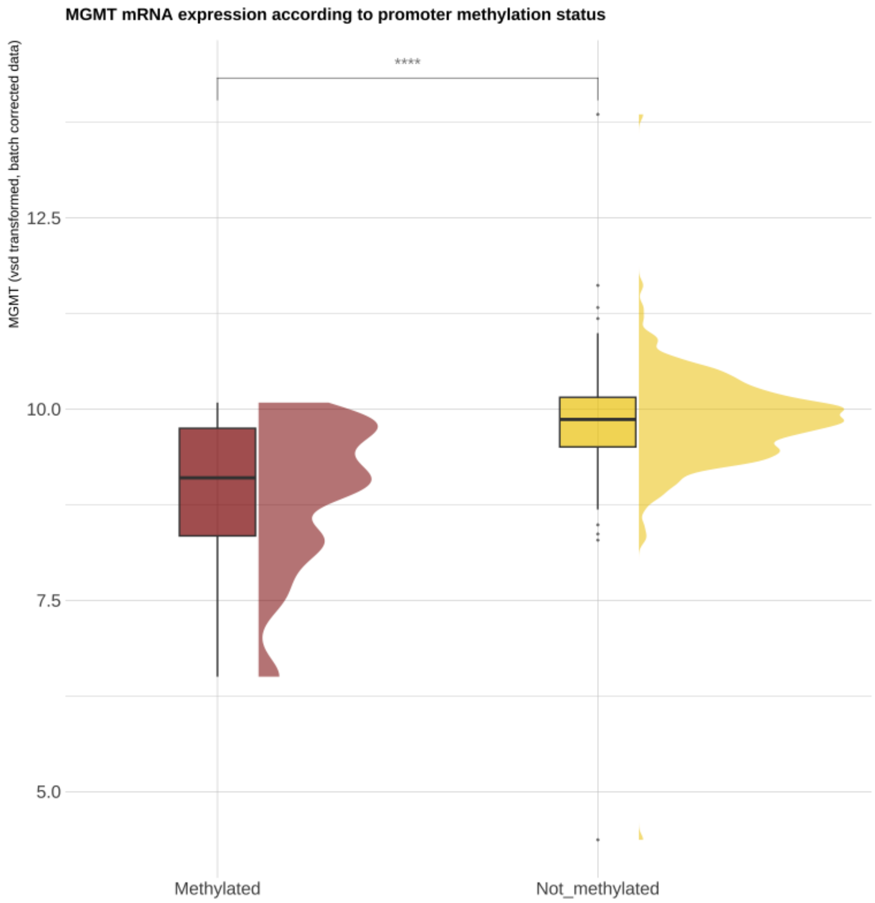

Supplementary Fig 3

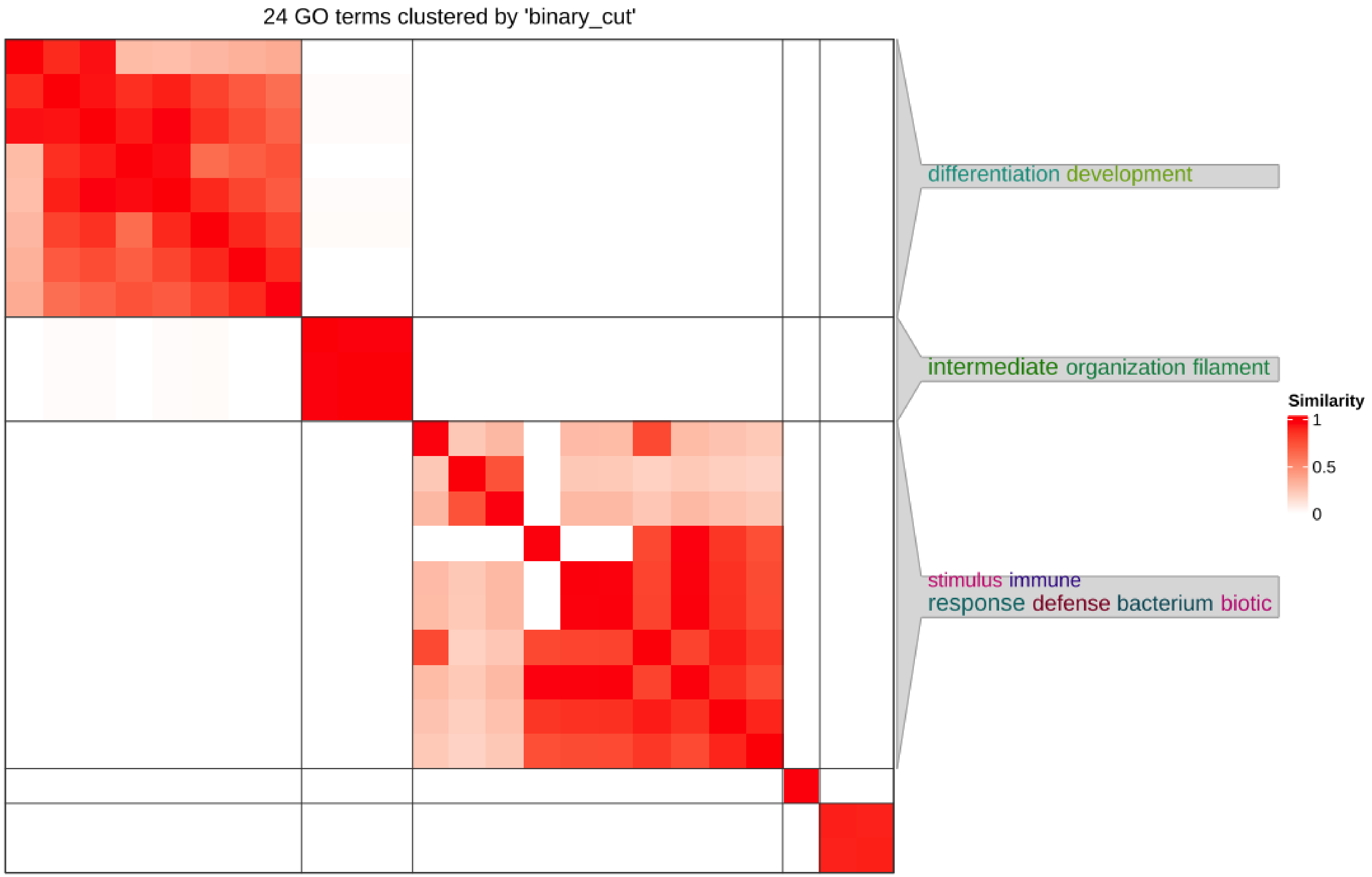

Supplementary Fig 4

Cell types enrichment analysis in MGMT methylated vs -not methylated pancreatic cancers

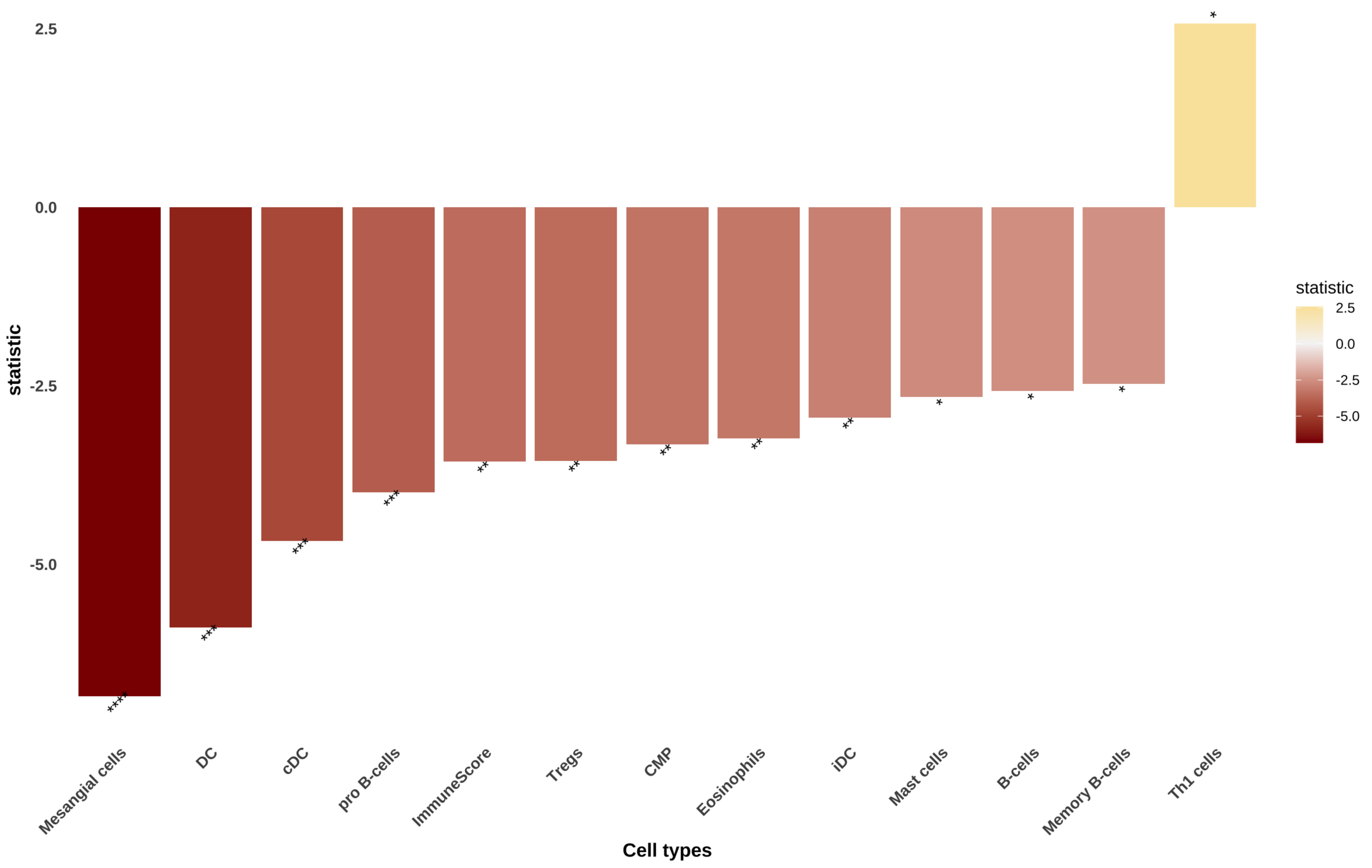

Supplementary Fig 5

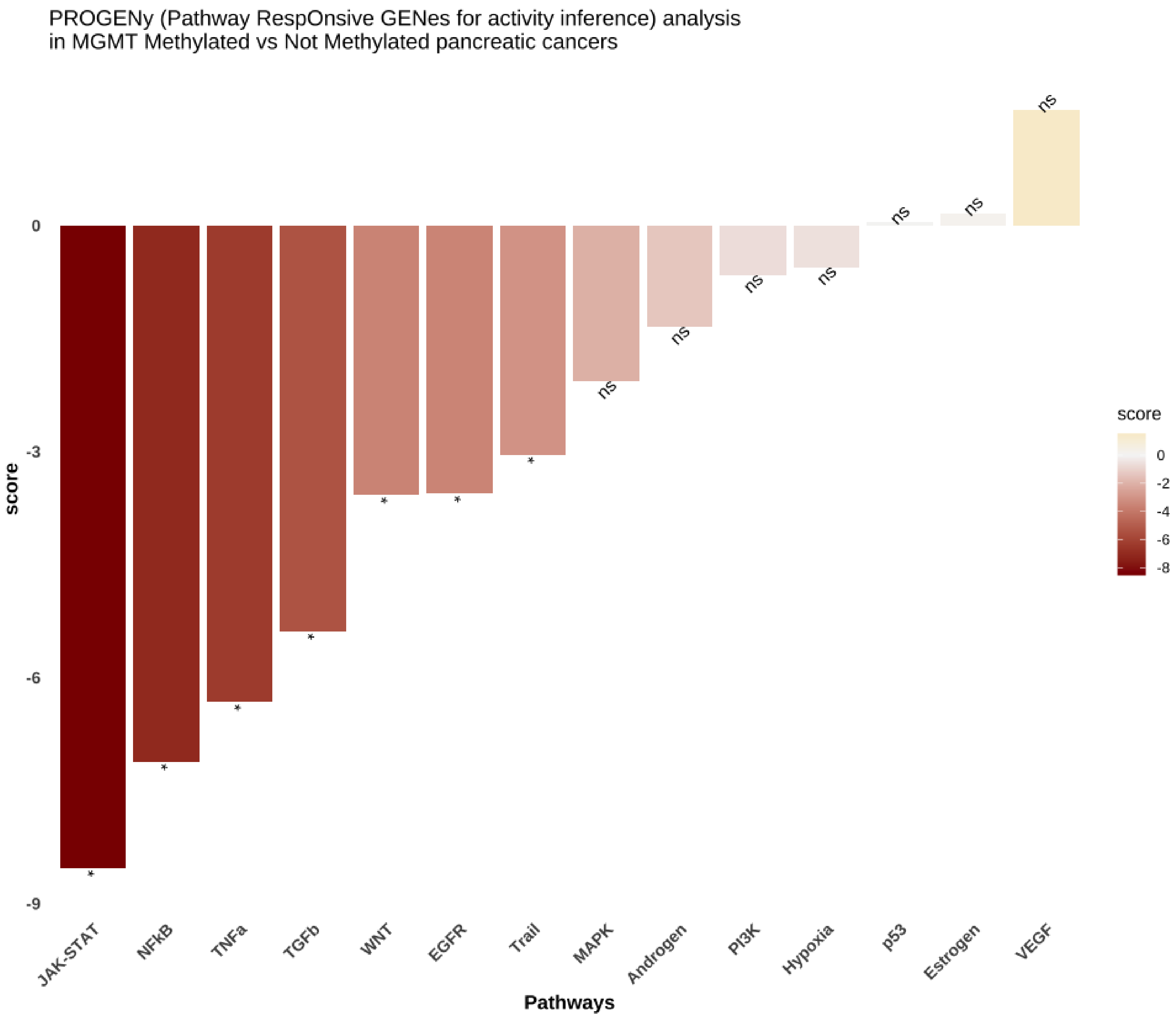

Supplementary Fig 6

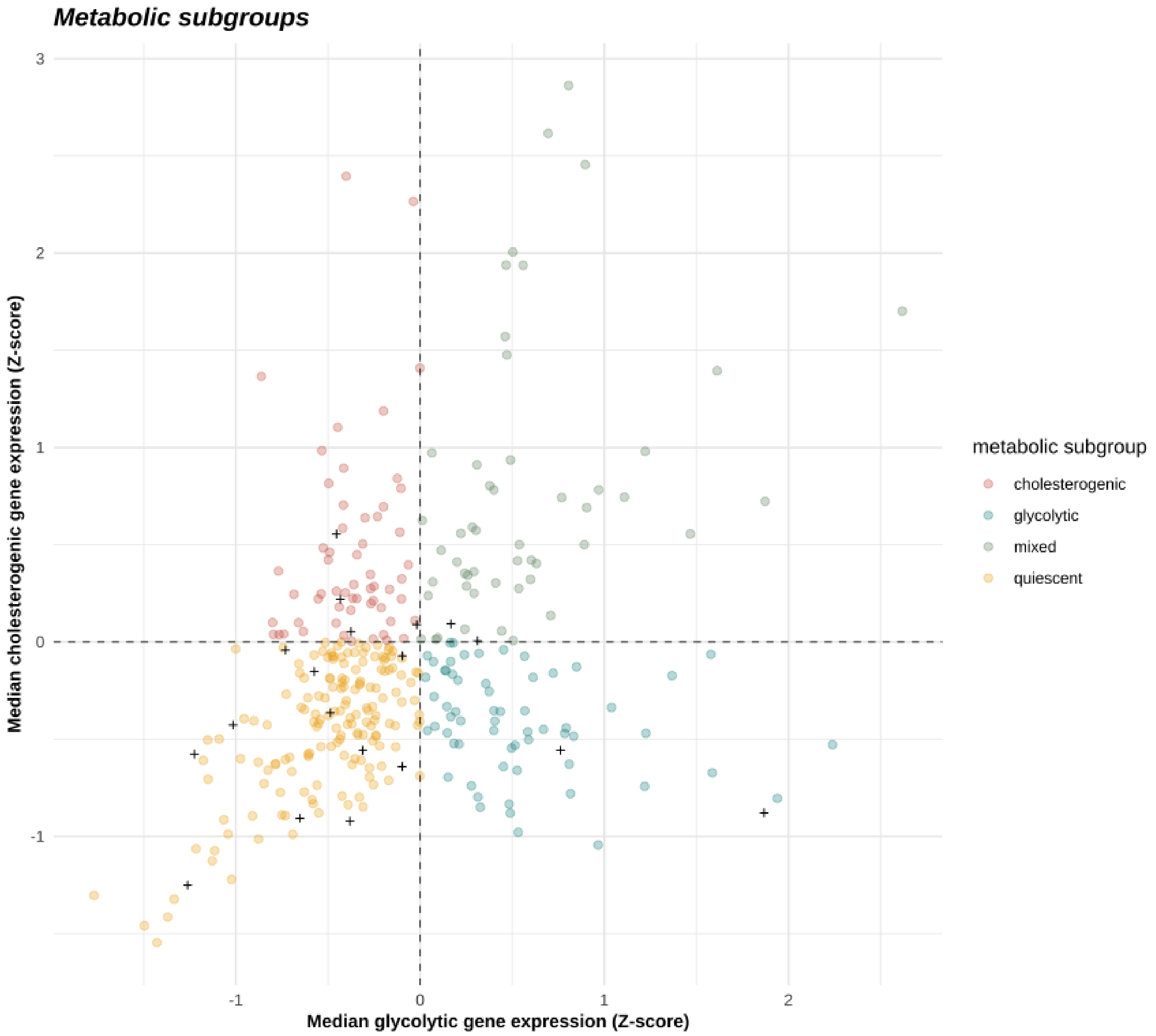

Supplementary Fig 7a-b

s7a

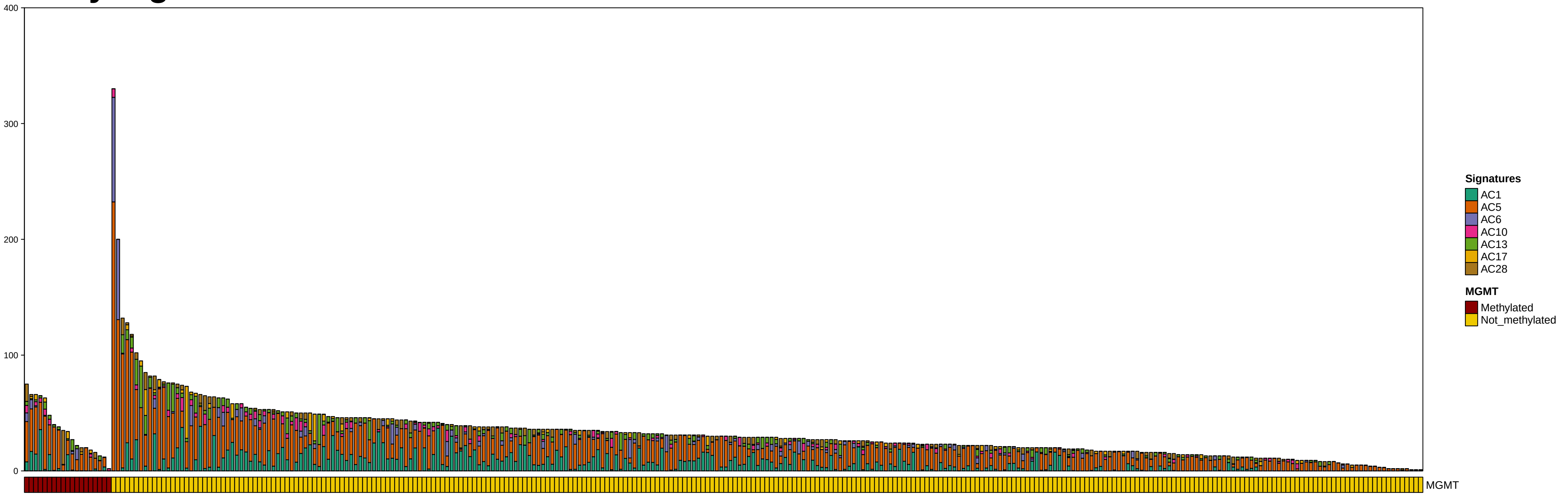

s7b

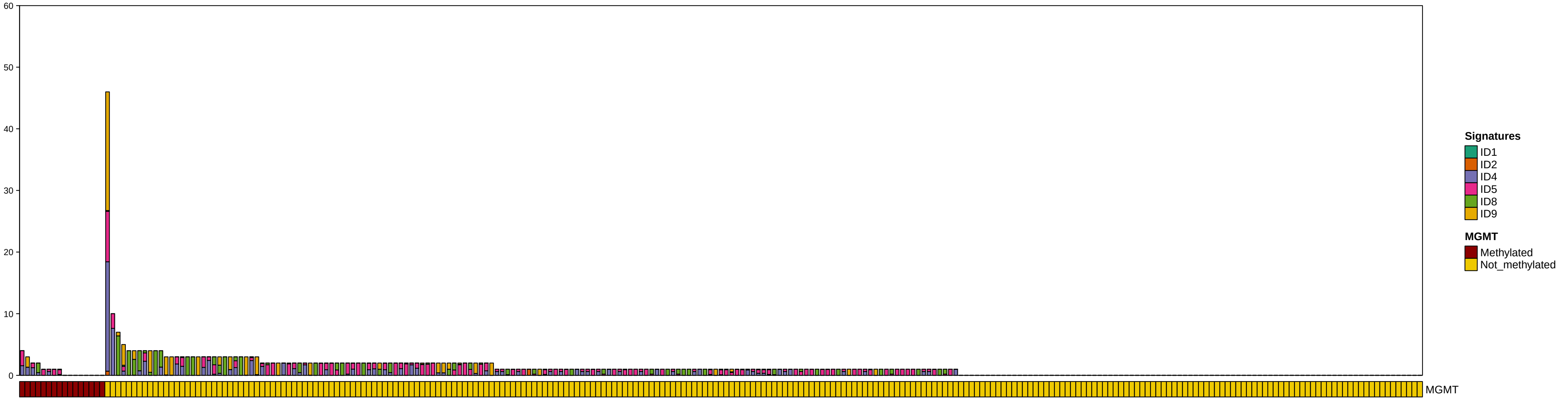

Supplementary Fig 7c-d

s7c

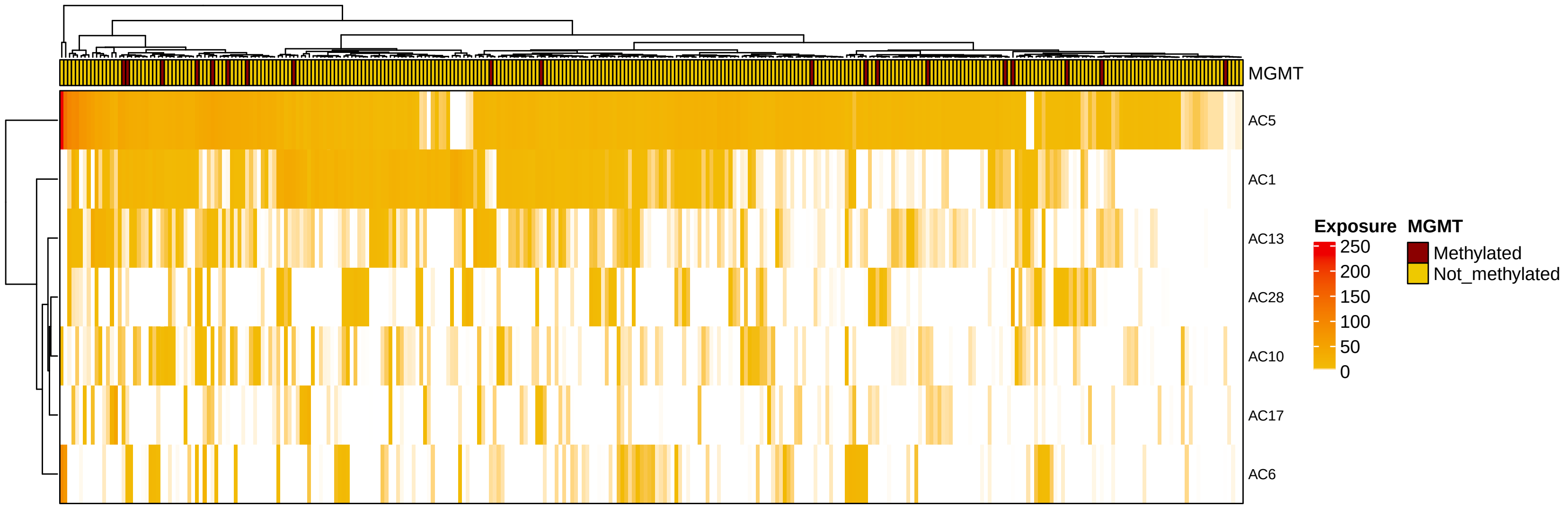

s7d

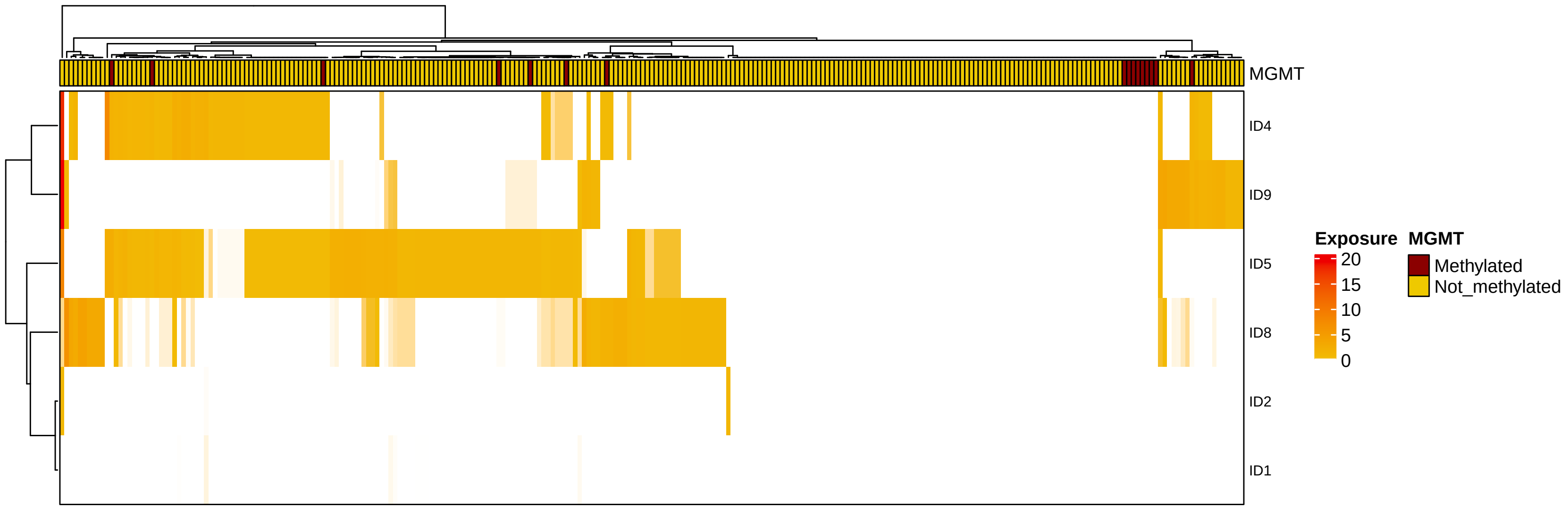

Supplementary Fig 8

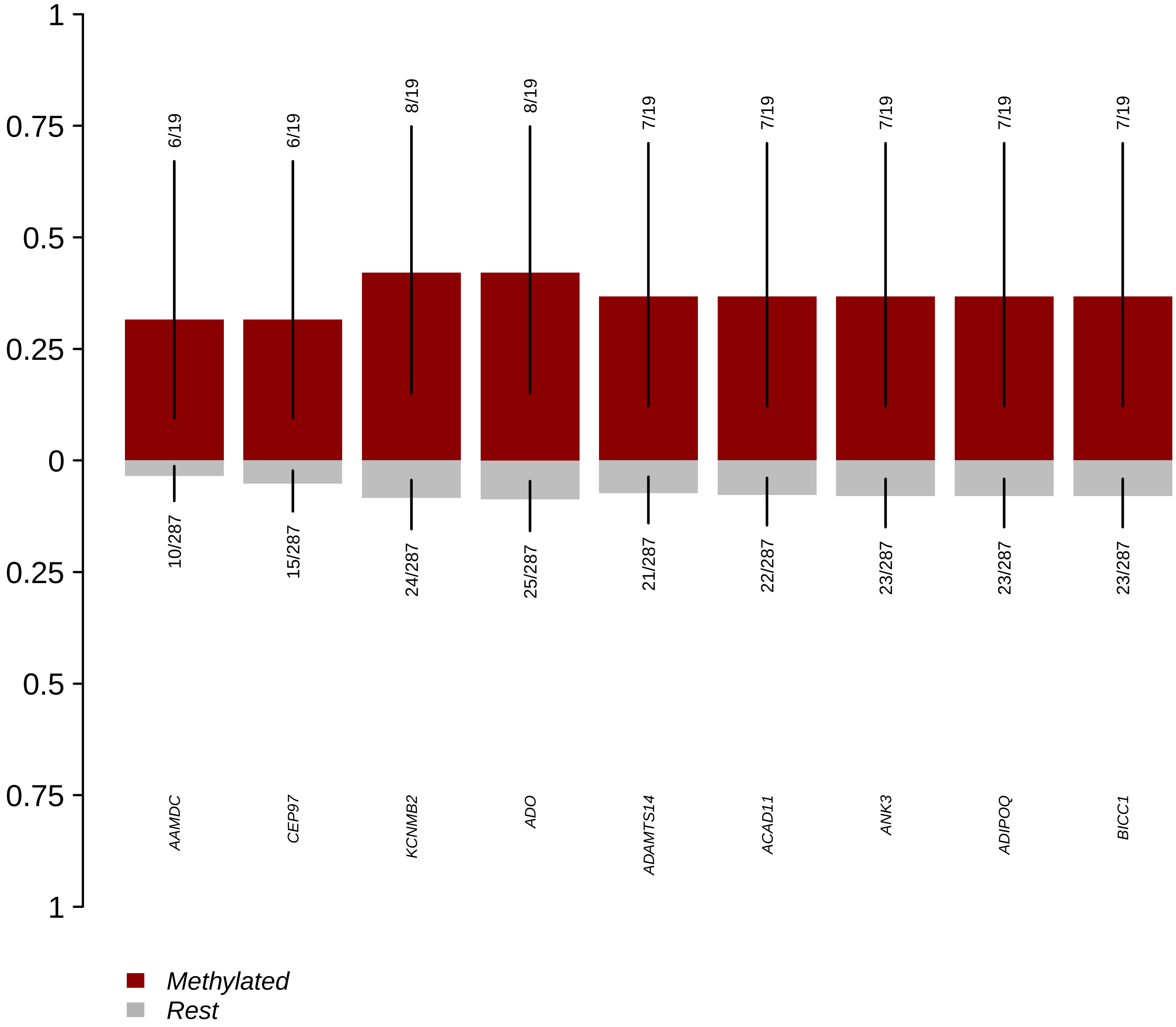

Supplementary Fig 9a-b

s9a

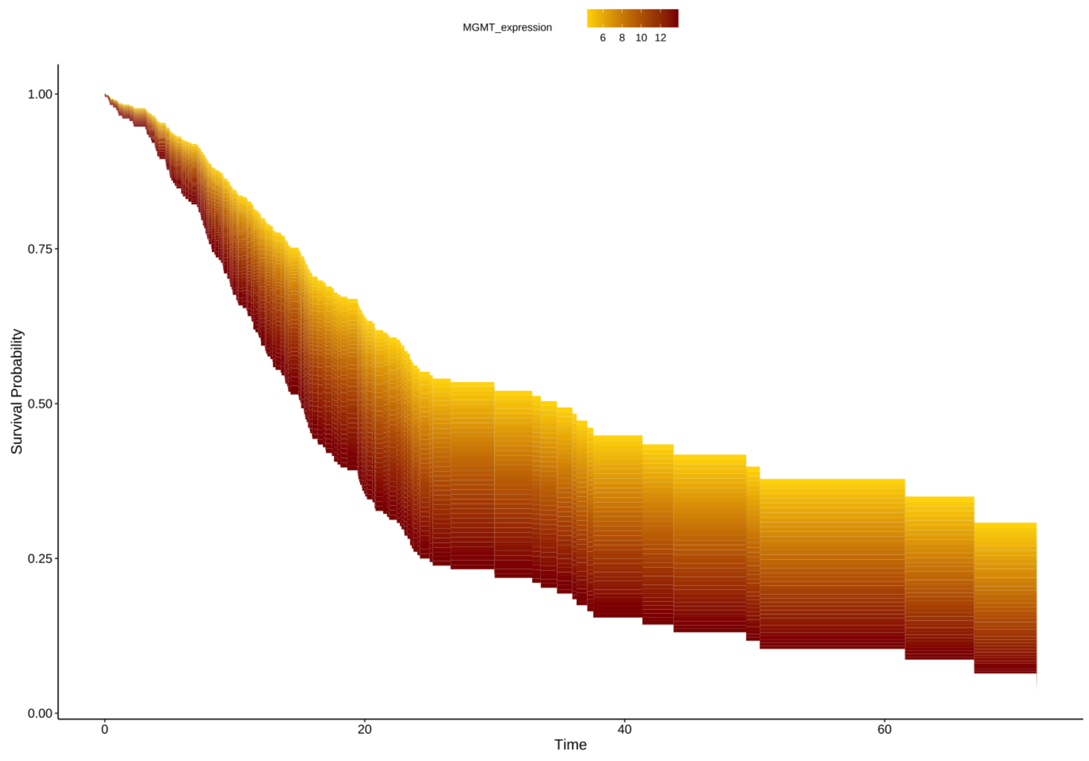

s9b

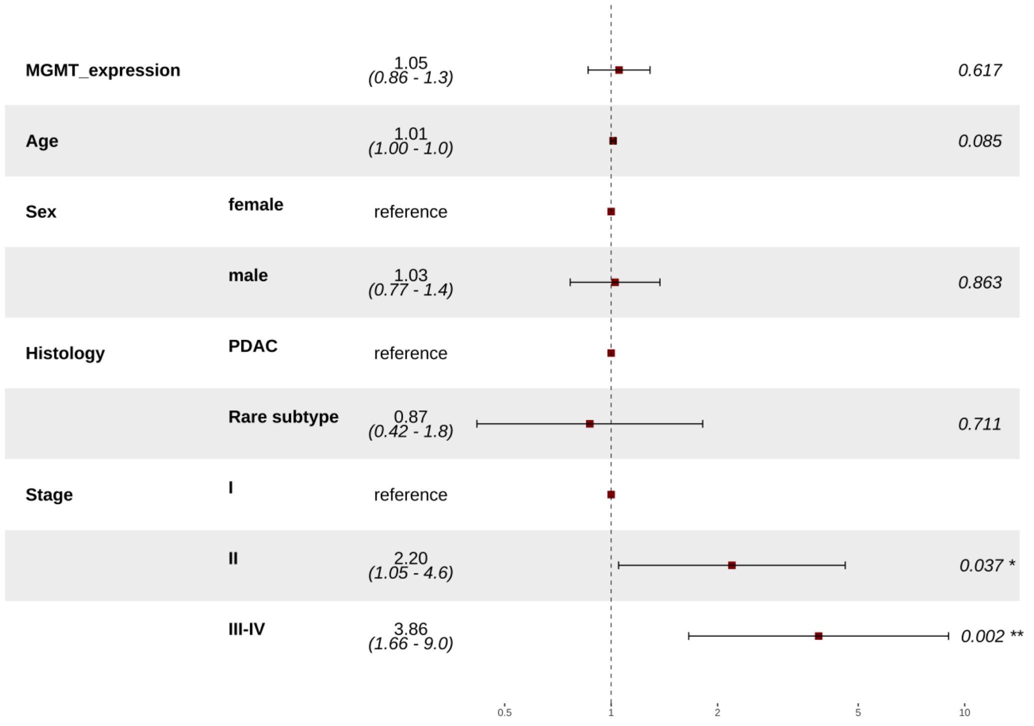

Supplementary Fig 10

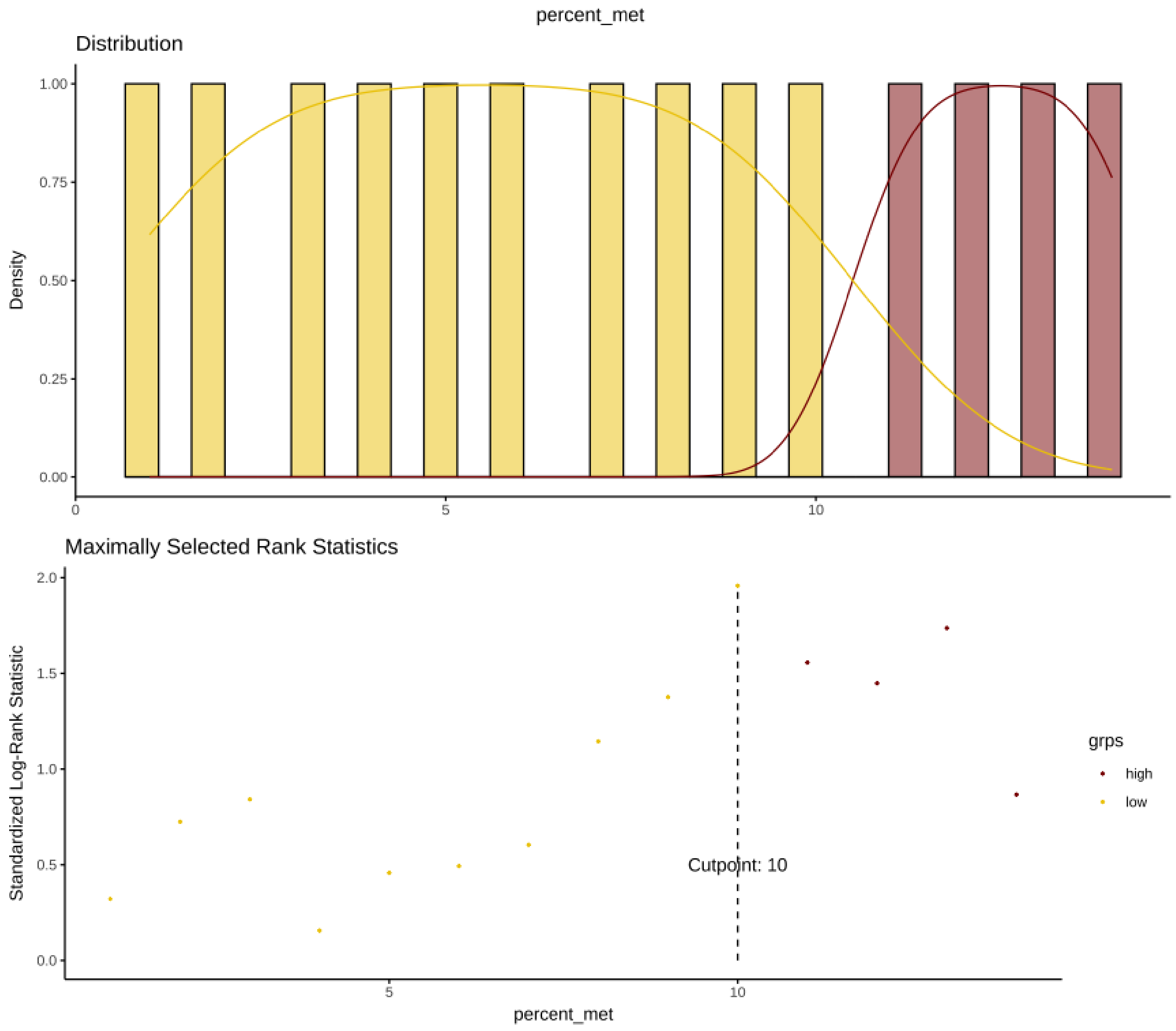

Supplementary Fig 11a-b

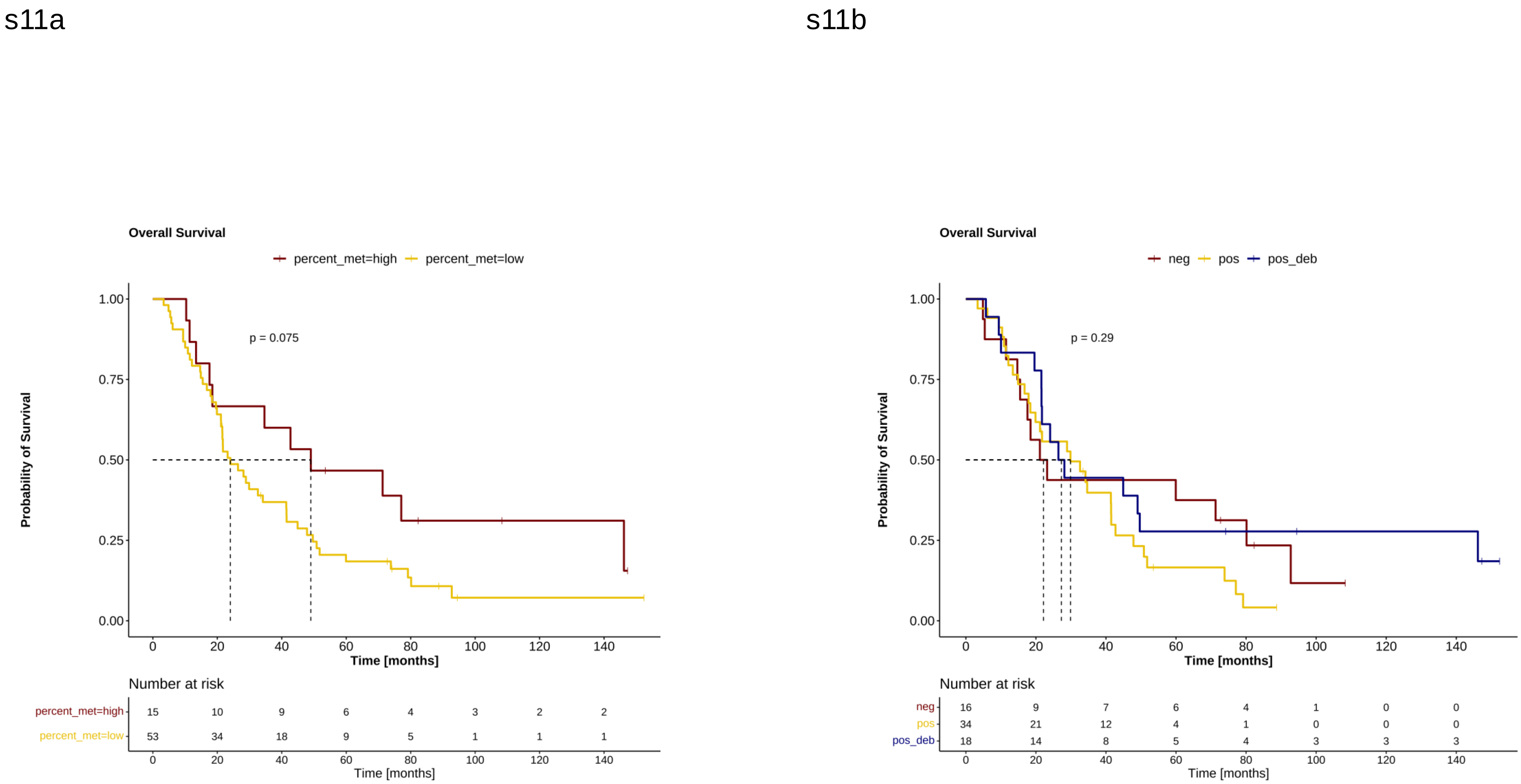

Supplementary Fig 12

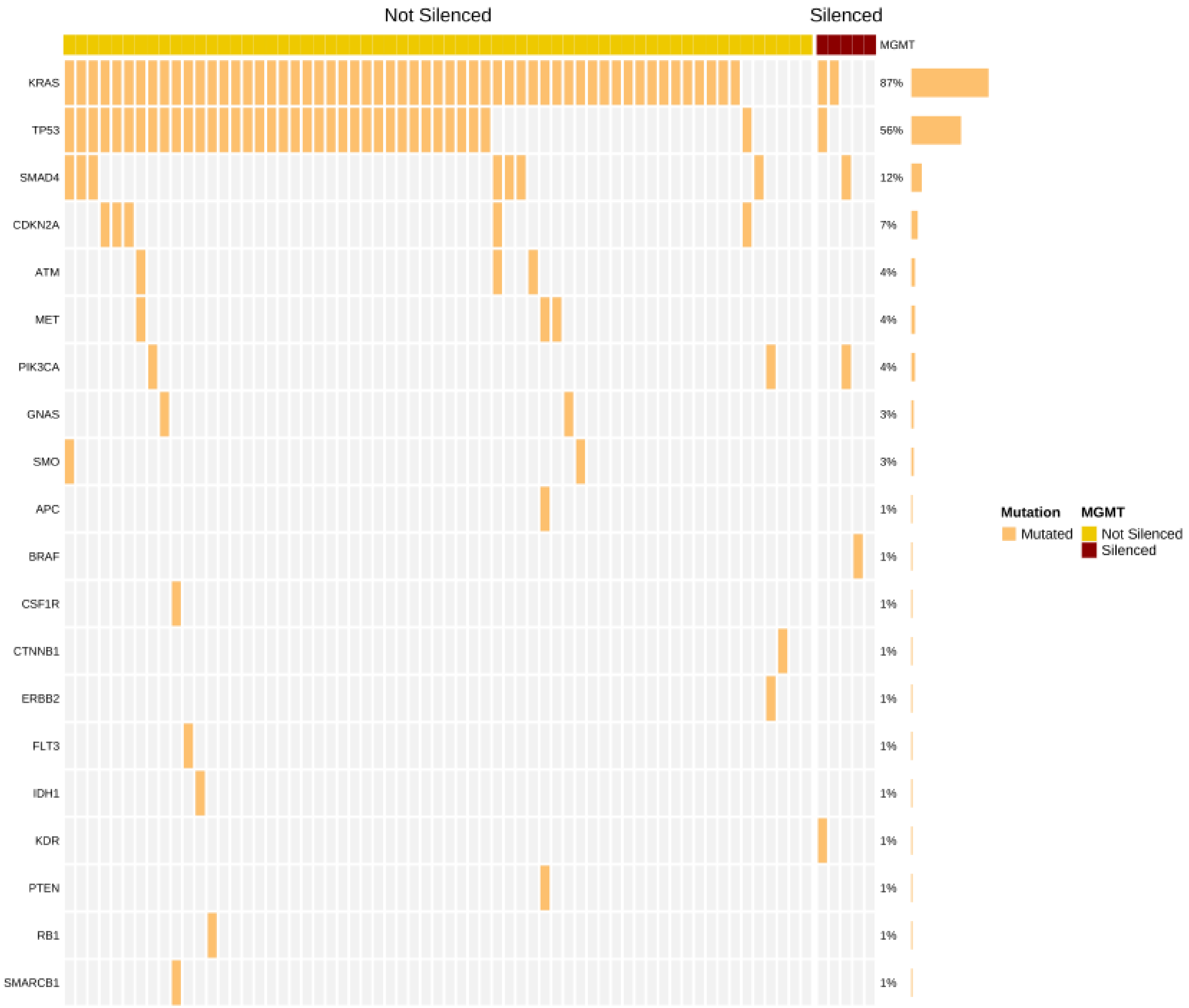

Supplementary Fig 13

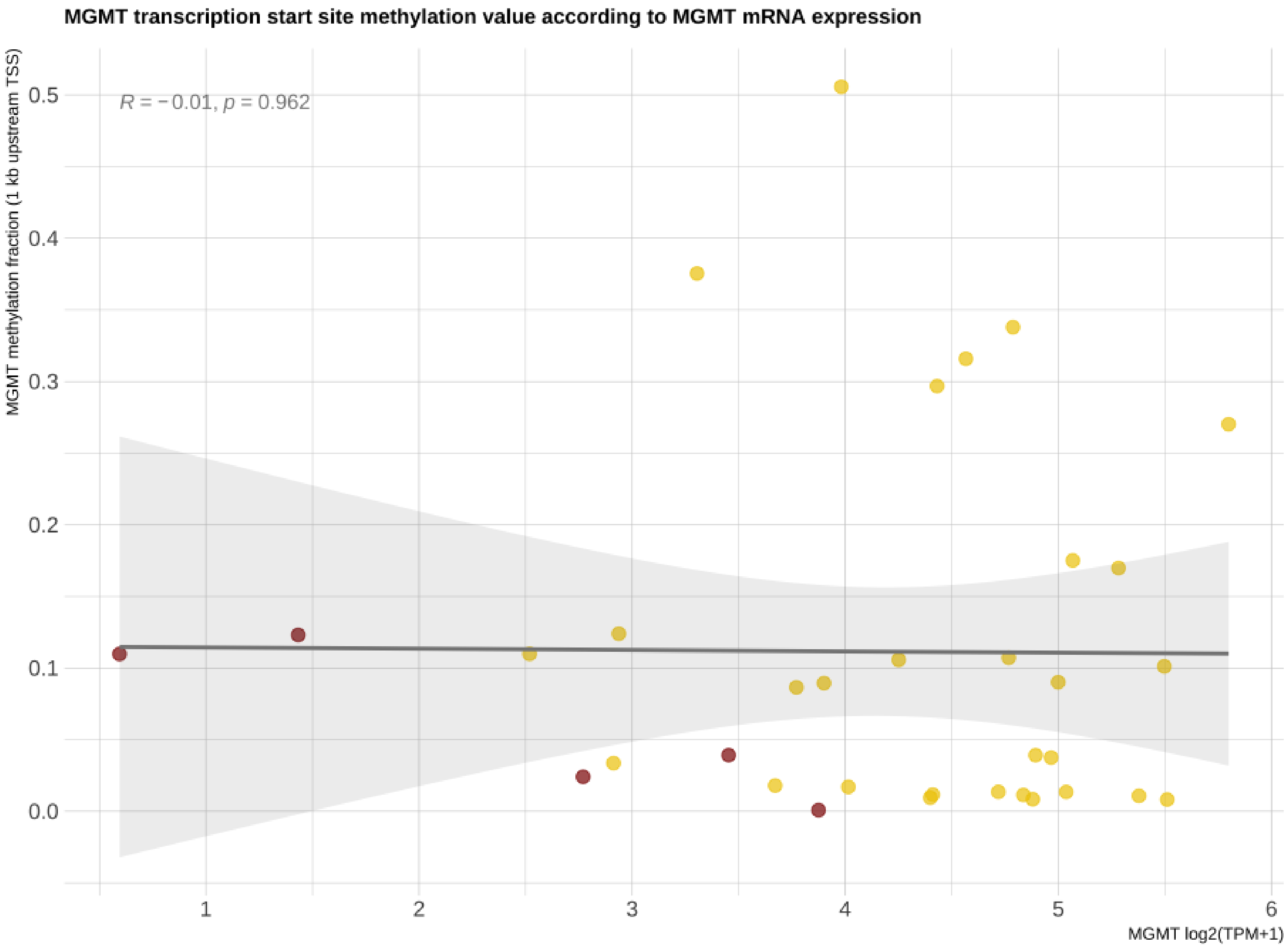

Supplementary Fig 14a-b

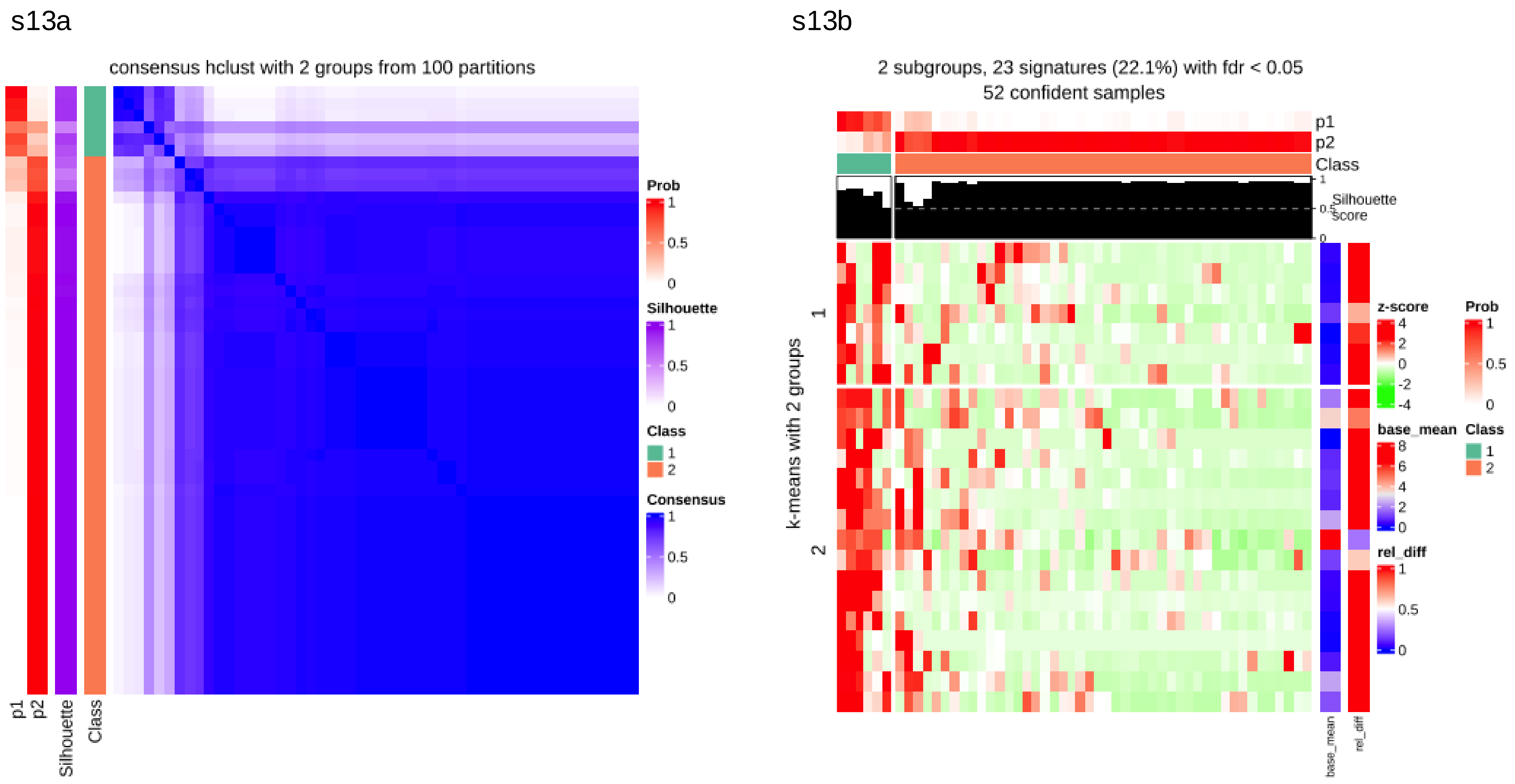

Supplementary Fig 14c

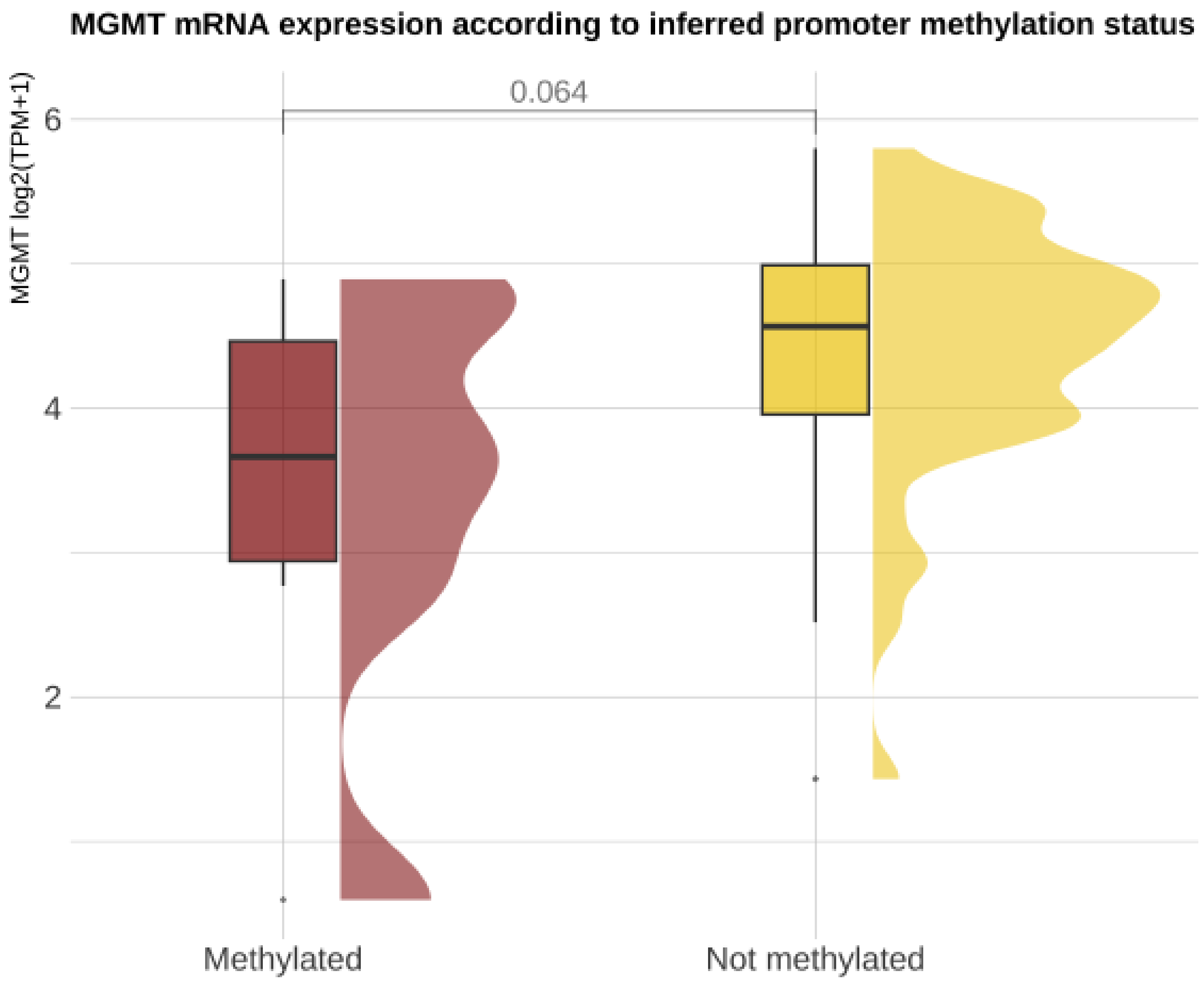

Supplementary Fig 15

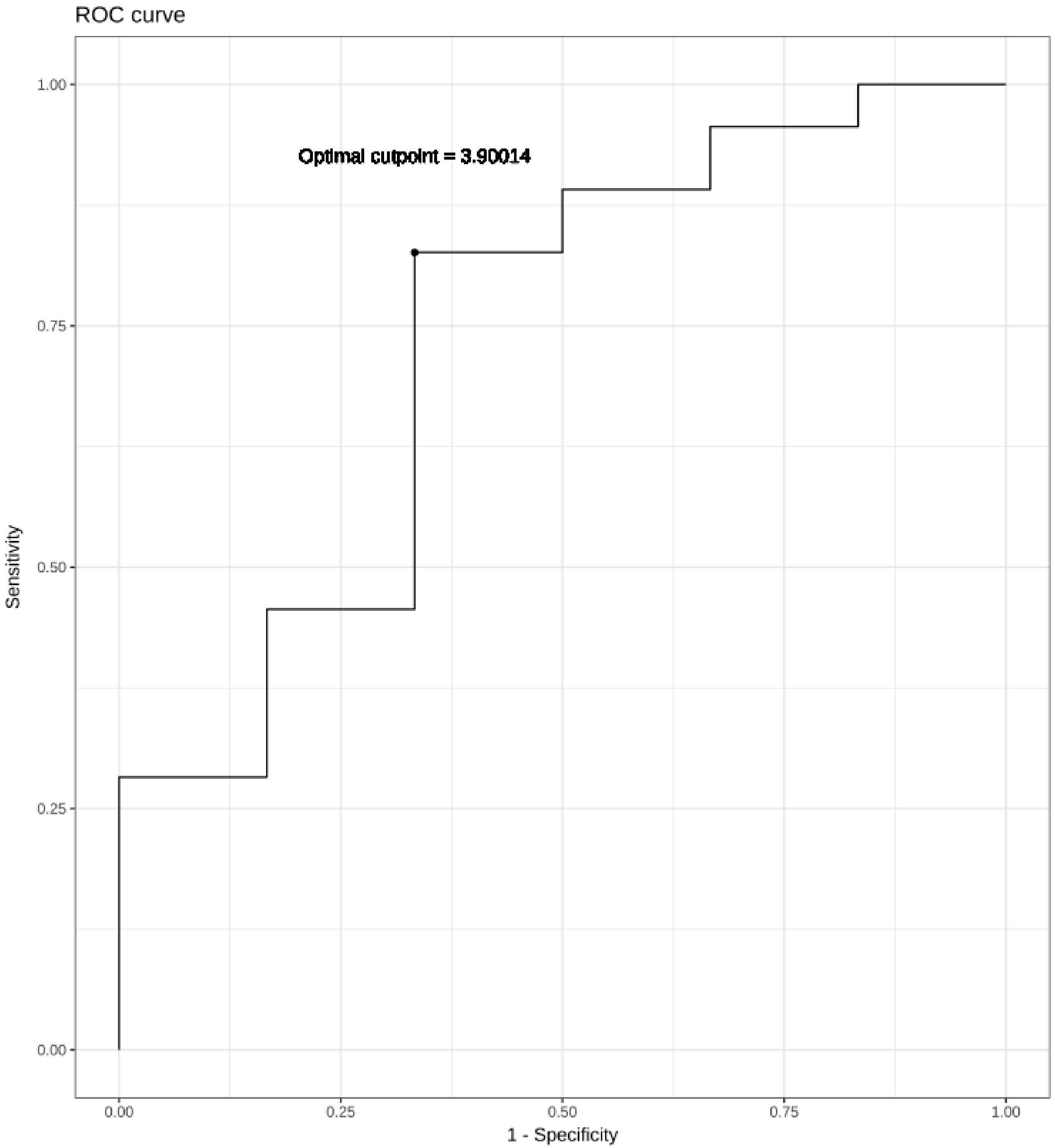

Supplementary Fig 16

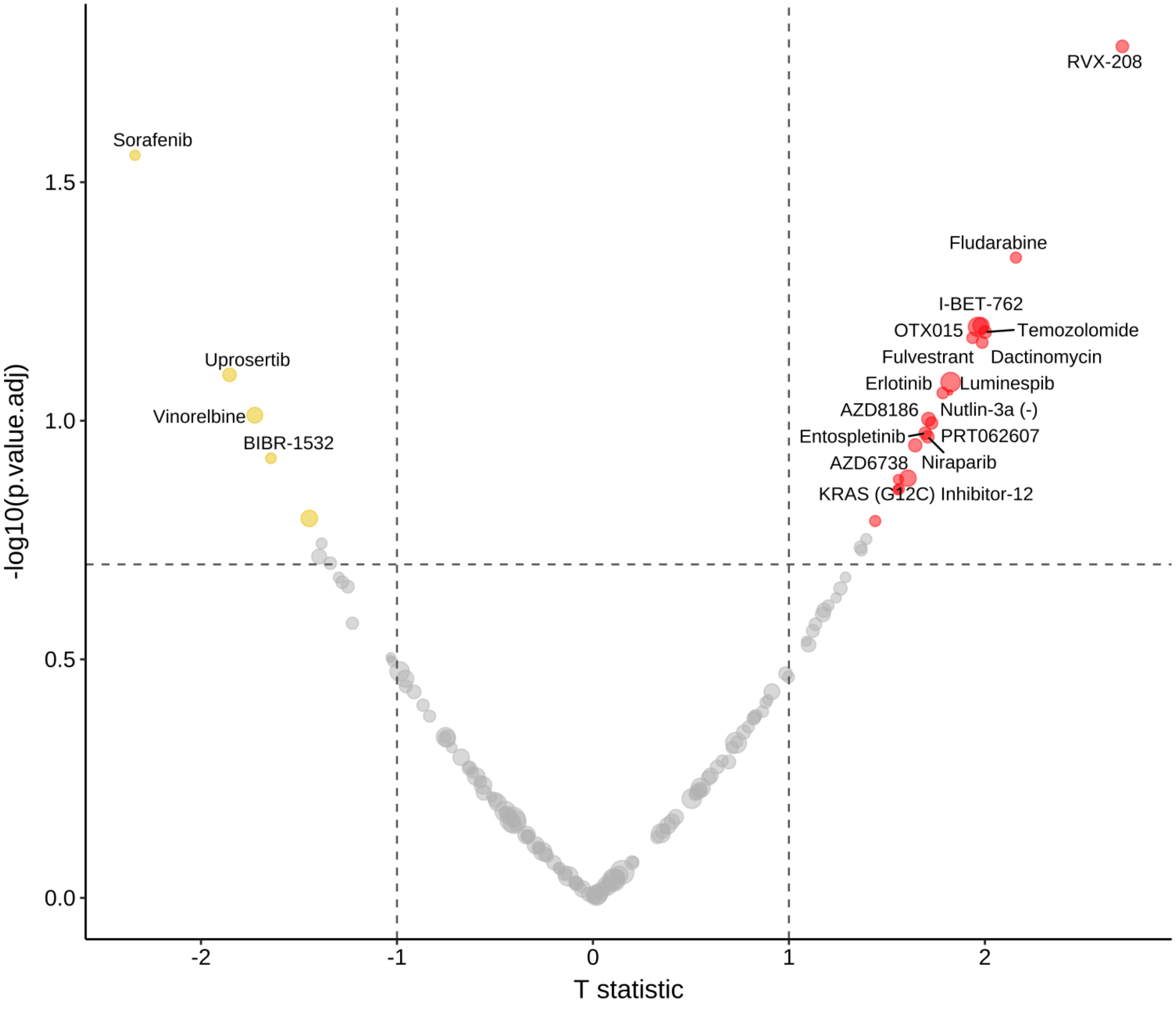

Supplement: Supplementary file 1 — Figure S1. MGMT methylation in PAC analysis methods pipeline. Genome methylation data from three cohorts (TCGA, CPTAC‐3, and PACA‐AU, for a total of 331 cases) were analyzed to identify MGMT‐methylated cases. Clinical, genomic, and transcriptomic data were then explored and compared according to this new classification. Then, a cohort of 69 PAC patients treated and profiled for MGMT status with pyrosequencing and IHC and with targeted panel NGS at Fondazione IRCCS Istituto Nazionale dei Tumori was explored to validate previous findings; in this cohort, clinical and genomic data according to MGMT status were compared. CPTAC‐3, Clinical Proteomic Tumor Analysis Consortium—cohort 3; IHC, Immunohistochemistry; MGMT, O6‐methylguanine‐DNA methyltransferase; NGS, Next Generation Sequencing; PAC, Pancreatic Cancer; PACA‐AU, Pancreatic Cancer Australia; TCGA, The Cancer Genome Atlas Program. Figure S2. (a) Correlogram showing correlation between MGMT expression values (vst) and beta‐ and M‐values of CpG islands located in the MGMT promoter region. While most CpGs had a weak, negative correlation with MGMT expression, the cg12434587 and cg12981137, included in MGMT‐STP27 had a significant, negative correlation with MGMT expression. The size of each ellipsis in the plot corresponds to the strength of the correlation. (b) Boxplots comparing MGMT expression (vst values) according to MGMT‐methylation status as defined by the MGMT‐STP27 algorithm. Density plots located on the right of the boxplots report the frequency of cases for each expression value. Wilcoxon mean rank‐sum p values are shown.MGMT, O6‐methylguanine‐DNA methyltransferase. Figure S3. Heatmap with word cloud annotation of clustered Gene Ontology terms from GSEA of differentially expressed genes between MGMT‐methylated versus not‐methylated PAC cases. Enrichment is done on keywords compared to Gene Ontology background vocabulary and the significance corresponds to the font size of the keywords.GO Terms, Gene Ontolog [file CAM4-13-e70393-s003.pdf]
