# Supplementary material for: Molecular Characterization and Clinical Relevance of MGMT‐Silenced Pancreatic Cancer
Source: Cancer Med. 2024 Dec 2;13(23):e70393. doi: 10.1002/cam4.70393 (PMC11609587; doi:10.1002/cam4.70393)
Supplement: Supplementary file 2 — Table S1. [file CAM4-13-e70393-s002.docx]

**Supplementary Table 1.** Drug sensitivity according to MGMT expression. The table reports results of the linear regression analysis testing the impact of MGMT expression [log2(tpm+1 values), dichotomized according to the optimal cutoff to distinguish inferred-*MGMT* status as in **Supplementary Figure 15**] on AUC values of anticancer compounds tested within the GDSC project. Higher T statistic values correspond to drugs that have higher AUC (i.e. lower sensitivity) with high MGMT expression values. Adjusted (after Benjamini–Hochberg multiple test correction) p value are reported.

Abbreviations: AUC: area under the curve; MGMT: O^6^-methylguanine-DNA methyltransferase.

| **Compound** | **AUC,**  **MGMT-not methylated** | **AUC,**  **MGMT-methylated** | **Estimate** | **Standard error** | **T statistic** | **p value** |
| --- | --- | --- | --- | --- | --- | --- |
| sorafenib (GDSC2:1085) | 0,933 | 0,951 | -0,025 | 0,011 | -2,337 | 0,028 |
| fludarabine (GDSC2:1813) | 0,988 | 0,928 | 0,026 | 0,012 | 2,158 | 0,046 |
| I-BET-762 (GDSC2:1624) | 0,964 | 0,921 | 0,056 | 0,028 | 1,980 | 0,063 |
| OTX015 (GDSC2:1626) | 0,950 | 0,888 | 0,082 | 0,042 | 1,964 | 0,064 |
| temozolomide (GDSC2:1375) | 0,982 | 0,930 | 0,032 | 0,016 | 2,001 | 0,065 |
| fulvestrant (GDSC2:1200) | 0,967 | 0,947 | 0,011 | 0,006 | 1,936 | 0,068 |
| uprosertib (GDSC2:2106) | 0,881 | 0,993 | -0,034 | 0,019 | -1,855 | 0,080 |
| AUY922 (GDSC2:1559) | 0,945 | 0,729 | 0,079 | 0,043 | 1,825 | 0,083 |
| erlotinib (GDSC2:1168) | 0,966 | 0,869 | 0,023 | 0,013 | 1,785 | 0,087 |
| vinorelbine (GDSC2:2048) | 0,969 | 0,911 | -0,045 | 0,026 | -1,725 | 0,097 |
| AZD8186 (GDSC2:1918) | 0,958 | 0,821 | 0,032 | 0,019 | 1,712 | 0,099 |
| PRT062607 (GDSC2:1631) | 0,934 | 0,919 | 0,026 | 0,015 | 1,728 | 0,101 |
| GS-9973 (GDSC2:1630) | 0,910 | 0,935 | 0,026 | 0,016 | 1,696 | 0,106 |
| niraparib (GDSC2:1177) | 0,985 | 0,933 | 0,026 | 0,015 | 1,709 | 0,108 |
| AZD6738 (GDSC2:1917) | 0,944 | 0,850 | 0,028 | 0,017 | 1,645 | 0,113 |
| alpelisib (GDSC2:1560) | 0,942 | 0,933 | 0,016 | 0,010 | 1,559 | 0,133 |
| gefitinib (GDSC2:1010) | 0,924 | 0,920 | 0,017 | 0,011 | 1,563 | 0,139 |
| VX-11e (GDSC2:2096) | 0,887 | 0,928 | -0,042 | 0,029 | -1,447 | 0,160 |
| osimertinib (GDSC2:1919) | 0,958 | 0,936 | 0,017 | 0,012 | 1,440 | 0,162 |
| olaparib (GDSC2:1017) | 0,993 | 0,943 | 0,017 | 0,012 | 1,395 | 0,177 |
| ML323 (GDSC2:1629) | 0,908 | 0,935 | -0,016 | 0,012 | -1,384 | 0,181 |
| WIKI4 (GDSC2:1940) | 0,987 | 0,843 | 0,022 | 0,016 | 1,366 | 0,184 |
| GSK2606414 (GDSC2:1618) | 0,983 | 0,898 | 0,018 | 0,013 | 1,370 | 0,187 |
| BVD-523 (GDSC2:2047) | 0,954 | 0,955 | -0,021 | 0,016 | -1,251 | 0,223 |
| BMS-345541 (GDSC2:1249) | 0,937 | 0,894 | 0,023 | 0,018 | 1,263 | 0,225 |
| cyclophosphamide (GDSC2:1512) | 0,956 | 0,977 | 0,013 | 0,010 | 1,241 | 0,235 |
| mirin (GDSC2:1048) | 0,951 | 0,945 | 0,015 | 0,013 | 1,201 | 0,244 |
| AZ6102 (GDSC2:2109) | 0,897 | 0,834 | 0,025 | 0,021 | 1,179 | 0,250 |
| entinostat (GDSC2:1593) | 0,917 | 0,908 | 0,026 | 0,022 | 1,173 | 0,255 |
| AZD8931 (GDSC2:1549) | 0,910 | 0,879 | 0,018 | 0,016 | 1,136 | 0,267 |
| SCH900776 (GDSC2:2046) | 0,904 | 0,904 | 0,018 | 0,016 | 1,122 | 0,276 |
| AMG-319 (GDSC2:2045) | 0,966 | 0,945 | -0,010 | 0,009 | -1,031 | 0,314 |
| paclitaxel (GDSC2:1080) | 0,965 | 0,982 | -0,010 | 0,010 | -1,022 | 0,320 |
| BI-97C1 (GDSC2:1849) | 0,802 | 0,810 | -0,043 | 0,043 | -0,986 | 0,335 |
| NVP-BEZ235 (GDSC2:1057) | 0,854 | 0,858 | -0,030 | 0,031 | -0,958 | 0,347 |
| bortezomib (GDSC2:1191) | 0,975 | 0,779 | 0,024 | 0,026 | 0,913 | 0,370 |
| BMS-536924 (GDSC2:1091) | 0,881 | 0,838 | -0,018 | 0,019 | -0,913 | 0,370 |
| PCI-34051 (GDSC2:1621) | 0,953 | 0,965 | 0,010 | 0,011 | 0,893 | 0,385 |
| AZD4547 (GDSC2:1786) | 0,998 | 0,933 | 0,011 | 0,013 | 0,882 | 0,390 |
| lapatinib (GDSC2:1558) | 0,938 | 0,948 | -0,013 | 0,015 | -0,867 | 0,395 |
| tamoxifen (GDSC2:1199) | 0,976 | 0,884 | 0,012 | 0,015 | 0,830 | 0,415 |
| Sinularin (GDSC2:1838) | 0,947 | 0,975 | -0,012 | 0,014 | -0,834 | 0,416 |
| MIM-1 (GDSC2:1996) | 0,994 | 0,929 | 0,013 | 0,016 | 0,825 | 0,418 |
| GSK2110183C (GDSC2:1912) | 0,946 | 0,857 | 0,014 | 0,017 | 0,819 | 0,421 |
| GDC 0068 (GDSC2:1924) | 0,939 | 0,954 | -0,010 | 0,013 | -0,786 | 0,440 |
| oxaliplatin (GDSC2:1089) | 0,968 | 0,949 | 0,006 | 0,008 | 0,772 | 0,448 |
| GDC-0941 (GDSC2:1058) | 0,853 | 0,812 | 0,016 | 0,020 | 0,769 | 0,449 |
| GNE317 (GDSC2:1926) | 0,807 | 0,744 | -0,024 | 0,032 | -0,745 | 0,463 |
| venetoclax (GDSC2:1909) | 0,999 | 0,908 | 0,010 | 0,014 | 0,735 | 0,471 |
| gemcitabine (GDSC2:1190) | 0,893 | 0,608 | 0,037 | 0,051 | 0,730 | 0,473 |
| crizotinib (GDSC2:1083) | 0,976 | 0,965 | -0,008 | 0,011 | -0,720 | 0,484 |
| AZD5363 (GDSC2:1916) | 0,942 | 0,925 | 0,010 | 0,015 | 0,711 | 0,484 |
| CS-1421 (GDSC2:1564) | 0,880 | 0,928 | -0,020 | 0,029 | -0,673 | 0,507 |
| 5-fluorouracil (GDSC2:1073) | 0,936 | 0,865 | 0,009 | 0,014 | 0,659 | 0,516 |
| WEHI-539 (GDSC2:1997) | 0,975 | 0,898 | 0,012 | 0,019 | 0,634 | 0,532 |
| Pyridostatin (GDSC2:2044) | 0,951 | 0,928 | -0,008 | 0,013 | -0,632 | 0,533 |
| dabrafenib (GDSC2:1373) | 0,967 | 0,945 | -0,008 | 0,013 | -0,614 | 0,545 |
| camptothecin (GDSC2:1003) | 0,960 | 0,799 | 0,015 | 0,024 | 0,600 | 0,554 |
| navitoclax (GDSC2:1011) | 0,888 | 0,902 | -0,020 | 0,034 | -0,595 | 0,557 |
| taselisib (GDSC2:1561) | 0,908 | 0,887 | 0,013 | 0,021 | 0,590 | 0,561 |
| BDP-00009066 (GDSC2:1866) | 0,952 | 0,897 | -0,009 | 0,015 | -0,574 | 0,571 |
| Dihydrorotenone (GDSC2:1827) | 0,902 | 0,915 | -0,018 | 0,033 | -0,561 | 0,581 |
| MST-312 (GDSC2:1930) | 0,949 | 0,804 | 0,023 | 0,042 | 0,549 | 0,588 |
| AZD3759 (GDSC2:1915) | 0,949 | 0,935 | 0,004 | 0,008 | 0,542 | 0,593 |
| ibrutinib (GDSC2:1799) | 0,964 | 0,950 | 0,007 | 0,013 | 0,550 | 0,593 |
| BVD-523 (GDSC2:1908) | 0,905 | 0,832 | -0,013 | 0,025 | -0,536 | 0,597 |
| obatoclax (GDSC2:1068) | 0,914 | 0,855 | 0,013 | 0,024 | 0,532 | 0,600 |
| cediranib (GDSC2:1922) | 0,934 | 0,898 | 0,007 | 0,013 | 0,520 | 0,608 |
| mitoxantrone (GDSC2:1810) | 0,944 | 0,712 | 0,022 | 0,044 | 0,504 | 0,620 |
| PD-0325901 (GDSC2:1060) | 0,863 | 0,940 | -0,011 | 0,021 | -0,499 | 0,622 |
| foretinib (GDSC2:2040) | 0,847 | 0,794 | -0,015 | 0,030 | -0,486 | 0,631 |
| dinaciclib (GDSC2:1180) | 0,891 | 0,677 | -0,021 | 0,048 | -0,448 | 0,659 |
| PD-173074 (GDSC2:1049) | 0,964 | 0,968 | -0,006 | 0,013 | -0,452 | 0,662 |
| docetaxel (GDSC2:1007) | 0,947 | 0,867 | -0,008 | 0,018 | -0,440 | 0,664 |
| alisertib (GDSC2:1051) | 0,986 | 0,946 | 0,010 | 0,024 | 0,423 | 0,677 |
| ABT737 (GDSC2:1910) | 0,954 | 0,881 | -0,033 | 0,080 | -0,409 | 0,686 |
| NVP-ADW742 (GDSC2:1932) | 0,958 | 0,982 | -0,007 | 0,017 | -0,405 | 0,689 |
| cisplatin (GDSC2:1005) | 0,962 | 0,932 | -0,007 | 0,017 | -0,405 | 0,689 |
| vincristine (GDSC2:1818) | 0,970 | 0,730 | -0,025 | 0,064 | -0,400 | 0,694 |
| MK-1775 (GDSC2:1179) | 0,869 | 0,723 | 0,011 | 0,029 | 0,382 | 0,706 |
| dactinomycin (GDSC2:1911) | 0,839 | 0,542 | -0,021 | 0,056 | -0,371 | 0,714 |
| GSK1904529A (GDSC2:1093) | 0,972 | 0,903 | 0,004 | 0,010 | 0,368 | 0,716 |
| topotecan (GDSC2:1808) | 0,956 | 0,715 | 0,014 | 0,040 | 0,347 | 0,732 |
| uprosertib (GDSC2:1553) | 0,924 | 0,915 | 0,008 | 0,022 | 0,346 | 0,734 |
| teniposide (GDSC2:1809) | 0,982 | 0,854 | -0,012 | 0,034 | -0,338 | 0,739 |
| leflunomide (GDSC2:1578) | 0,920 | 0,872 | -0,005 | 0,016 | -0,333 | 0,743 |
| Elephantin (GDSC2:1835) | 0,950 | 0,915 | 0,005 | 0,016 | 0,328 | 0,747 |
| BPD-00008900 (GDSC2:1998) | 0,963 | 0,958 | -0,009 | 0,032 | -0,291 | 0,776 |
| WZ4003 (GDSC2:1614) | 0,984 | 0,967 | -0,003 | 0,011 | -0,279 | 0,784 |
| VE-822 (GDSC2:1613) | 0,993 | 0,948 | -0,005 | 0,017 | -0,279 | 0,785 |
| docetaxel (GDSC2:1819) | 0,950 | 0,595 | 0,017 | 0,065 | 0,263 | 0,796 |
| trametinib (GDSC2:1372) | 0,734 | 0,834 | -0,009 | 0,037 | -0,256 | 0,800 |
| irinotecan (GDSC2:1088) | 0,967 | 0,843 | -0,005 | 0,020 | -0,244 | 0,809 |
| buparlisib (GDSC2:1873) | 0,914 | 0,787 | -0,004 | 0,017 | -0,235 | 0,816 |
| Wee1 Inhibitor (GDSC2:1046) | 0,955 | 0,938 | 0,004 | 0,019 | 0,199 | 0,844 |
| vorinostat (GDSC2:1012) | 0,894 | 0,863 | -0,004 | 0,021 | -0,199 | 0,844 |
| linsitinib (GDSC2:1510) | 0,905 | 0,927 | -0,001 | 0,011 | -0,136 | 0,893 |
| pevonedistat (GDSC2:1529) | 0,997 | 0,751 | -0,006 | 0,044 | -0,126 | 0,901 |
| YM-155 (GDSC2:1941) | 0,379 | 0,301 | 0,005 | 0,044 | 0,108 | 0,915 |
| Podophyllotoxin bromide (GDSC2:1825) | 0,977 | 0,889 | 0,003 | 0,034 | 0,094 | 0,926 |
| palbociclib (GDSC2:1054) | 0,910 | 0,919 | -0,001 | 0,014 | -0,090 | 0,929 |
| YK-4-279 (GDSC2:1239) | 0,961 | 0,863 | -0,002 | 0,021 | -0,086 | 0,932 |
| MK-2206 (GDSC2:1053) | 0,850 | 0,908 | -0,001 | 0,016 | -0,083 | 0,934 |
| talazoparib (GDSC2:1259) | 0,944 | 0,842 | 0,002 | 0,023 | 0,066 | 0,948 |
| epirubicin (GDSC2:1511) | 0,932 | 0,784 | -0,002 | 0,029 | -0,053 | 0,958 |
| AZD7762 (GDSC2:1022) | 0,833 | 0,677 | 0,001 | 0,033 | 0,039 | 0,969 |
| PLX4720 (GDSC2:1036) | 0,979 | 0,929 | 0,000 | 0,011 | 0,036 | 0,971 |
| Oxyphenisatin acetate (GDSC2:1804) | 0,969 | 0,919 | 0,001 | 0,048 | 0,017 | 0,987 |
| cytarabine (GDSC2:1006) | 0,911 | 0,855 | 0,000 | 0,022 | 0,009 | 0,993 |
| XAV 939 (GDSC2:1268) | 0,979 | 0,918 | 0,000 | 0,017 | 0,007 | 0,994 |
