# Supplementary material for: Molecular Characterization and Clinical Relevance of MGMT‐Silenced Pancreatic Cancer
Source: Cancer Med. 2024 Dec 2;13(23):e70393. doi: 10.1002/cam4.70393 (PMC11609587; doi:10.1002/cam4.70393)
Supplement: Supplementary file 3 — Data S1. [file CAM4-13-e70393-s001.docx]

Molecular characterization and clinical relevance of

*MGMT*-silenced pancreatic cancer

**Running Title:** *MGMT*-silenced pancreatic cancer

Federico Nichetti^1,2*^, Marco Silvestri^3*^, Luca Agnelli^3^, Andrea Franza^1^, Chiara Pircher^1^, Simone Rota^1^, Paolo Ambrosini^1^, Giuseppe Fotia^1^, Jennifer Hüllein^2^, Giovanni Randon^1^, Panna Lajer^4^, Federica Perrone^3^, Elena Tamborini^3^, Giuseppe Leoncini^5^, Jorgelina Coppa^6^, Michele Droz Dit Busset^6^, Sara Pusceddu^1^, Massimo Milione^5^, Federica Morano^1^, Filippo Pietrantonio^1^, Maria Di Bartolomeo^1^, Giancarlo Pruneri^3,7^, Vincenzo Mazzaferro^6,7^, Daniel B. Lipka^4^, Bruno Christian Köhler^8,9,10^, Daniel Hübschmann^2,8,10^, Stefan Fröhling^8,10^, Filippo de Braud*^1,7^ and Monica Niger*^1^

^*^ These authors contributed equally to this work.

**Affiliations:**

^1^ Medical Oncology Department, Fondazione IRCCS Istituto Nazionale dei Tumori di Milano, Milan, Italy

^2^ Computational Oncology, Molecular Precision Oncology Program, National Center for Tumor Diseases (NCT) and German Cancer Research Center (DKFZ), Heidelberg, Germany

^3^ Second Division of Pathology, Department of Diagnostic Innovation, Fondazione IRCCS Istituto Nazionale dei Tumori, Milan, Italy

^4^ Section of Translational Cancer Epigenomics, Division of Translational Medical Oncology, German Cancer Research Center (DKFZ) and National Center for Tumor Diseases (NCT), Heidelberg, Germany

^5^ First Division of Pathology, Department of Pathology and Laboratory Medicine, Fondazione IRCCS Istituto Nazionale dei Tumori, Milan, Italy

^6^ Hepato-Pancreato-Biliary Surgery and Liver Transplantation, Fondazione IRCCS Istituto Nazionale dei Tumori, Milan, Italy

^7^ Department of Oncology and Hemato-Oncology, University of Milan, Milan, Italy.

^8^ Division of Translational Medical Oncology, National Center for Tumor Diseases (NCT) Heidelberg, German Cancer Research Center (DKFZ), Heidelberg, Germany

^9^ Liver Cancer Center Heidelberg, University Hospital Heidelberg, Heidelberg, Germany

^10^ German Cancer Consortium (DKTK), Heidelberg, Germany

^11^ Pattern Recognition and Digital Medicine Group, Heidelberg Institute for Stem cell Technology and Experimental Medicine (HI-STEM), Heidelberg, Germany

# SUPPLEMENTARY METHODS

## Public data acquisition

First, the Genomic Data Commons (GDC)^1^, the International Cancer Genome Consortium (ICGC)^2^ and the cBioPortal^3^ data portals were interrogated to identify eligible cohorts for this study. Three studies were identified, namely the TCGA PAAD (The Cancer Genome Atlas pancreatic cancer, n = 178)^4^, the CPTAC-3 (Clinical Proteomic Tumor Analysis Consortium 3, n = 69)^5^ and the PACA-AU (Australian Pancreatic Cancer Genome Initiative, n = 84)^6^, and only cases with available genomic, transcriptomic and methylation data were included. Molecular data together with available clinical annotations of the TCGA and CPTAC-3 cohorts were downloaded on June 1^st^ 2022 from the GDC Legacy Archive via the *TCGAbiolinks*^7^ R package, while PACA-AU data were retrieved and assembled from ICGC (Release 28, processed as of March 27, 2019, <https://dcc.icgc.org/>). Patients and tumor characteristics were double checked with supplementary material of the original papers and with cBioPortal (<https://www.cbioportal.org>) data. Tumor-only data were collected and analyzed, removing normal tissue samples.

## Methylation data and *MGMT* promoter methylation status definition

Illumina Human Methylation 450k raw data were preprocessed to obtain M-values and analyzed by means of a logistic regression model using the *mgmtstp27*^8^ package. In detail, the model predicts *MGMT* promoter methylation status according to two key CpG probes (cg12434587 and cg12981137) located in the *MGMT* promoter that were previously identified to be functionally relevant. As a further validation, Pearson correlation scores between beta- and M- values of CpG islands located on the *MGMT* promoter and MGMT gene expression values were calculated. Once *MGMT* promoter methylation status was defined, MGMT gene expression values were compared between methylated and not-methylated samples.

## RNAseq data analysis

For RNAseq data, expression was used “as-is”, i.e. RNAseq data were not realigned and gene-level expression estimates were downloaded and analyzed in terms of raw counts, fragments per kilobase per million reads (FPKM, for PACA-AU) or transcripts per million (TPM, for TCGA and CPTAC-3), harmonized against GRCh37. Batch-corrected, variance-stabilizing transformed (vst) count data were used to compare MGMT expression with its promoter methylation status.

### Differential expression analysis, GSEA and PROGENy

Differential expression analysis was performed with *DESeq2*^9^ on count data, joint after batch correction by means of surrogate variable analysis^10^. Differential expression analysis results were used to generate a ranked gene list (with thresholds of adjusted p < 0.01 and log2FC > 0.58) and perform Gene Set Enrichment Analysis (GSEA) using the Gene Ontology (GO) Biological Processes resource with the *clusterProfiler^11^* package, and summarized using *simplifyEnrichment^12^*. Furthermore, the normalized weighted mean activity of 14 different signaling pathways, as inferred using the PROGENy^13,14^ algorithm, was compared between *MGMT*-methylated and not-methylated cases according to the ranked list of differentially expressed genes. Also, conservative (adjusted p < 0.005 and log2FC > 2.5) thresholds were applied to the list of differentially expressed genes as a “*MGMT*-silencing signature” to predict *MGMT* status from the tumor transcriptome of external samples.

### Gene expression-based subtyping

Previously defined transcriptomic-driven subtypes of PAC were inferred using a customized pipeline. In detail, gene signatures were obtained from the original publications^6,15,16^ and filtered for available genes in the study cohort. The TCGA gene-wise z-scored, adjusted (after removal of genes with more than 25% of cases with missing value or genes with <5^th^ percentile variance across the cohort and conversion of outliers to 5^th^ and 95^th^ percentile values) RNA expression matrices for these signatures (Moffitt N = 48, Collisson N = 60; Bailey N = 4082) underwent consensus clustering with *cola^17^* using ATC (ability to correlate) to extract features and spherical k-means clustering (skmeans) for subgroup classification, fixing the number of required subgroups (K) according to each original subtyping scheme and checking that these Ks performed as best according to silhouette scores, Jaccard index and Consensus Cumulative Distribution Function. Clustering results were then labeled according to gene expression patterns and by comparing obtained subgroups with already annotated molecular subtypes in the TCGA cohort. Then, for each transcriptomic subtype, the obtained TCGA signature centroid matrix was used to predict the subtypes in CPTAC-3 and PACA-AU samples by the Euclidean/cosine distance method.

In a separate analysis, metabolic subtypes were determined based on the median (Z-score transformed) expression of 14 glycolytic and 15 cholesterogenic genes, as previously described^15^. Obtained PAC subtypes were then tested for distribution according to *MGMT* status.

### Immune and stromal cells inference

Deconvolution methods were adopted to infer the proportion of immune and stromal cells in the tumor microenvironment. The ESTIMATE algorithm^18^ was used to derive the overall immune and stromal score for each sample. Moreover, two immune cell deconvolution methods were tested, namely xCell^19^ and TIMER^20^ (using a PAC-specific reference profile) and compared for robustness of results. For these methods, each individual cohort was analyzed separately using TPM values and pooled only thereafter.

## Genomic data

TCGA, CPTAC-3 and PACA-AU genomic data, in terms of single nucleotide variants (SNVs), insertion-deletions (indels) and copy number variations (CNVs, namely amplifications and deletions) were downloaded from cBioPortal and ICGC, respectively, and used “as-is”.

All the variants showing at least one of the following parameters were excluded: a) Depth of coverage < 50; b) Non coding regions; c) variant allele fraction (VAF) <= 5% or > 75%, d) FFPE artifact (VAF <= 10% & C>T or VAF <= 10% & G>A). Descriptive analysis and assessment of differentially mutated SNVs and indels between *MGMT*-methylated and not-methylated samples was performed using *maftools^21^.* Mutually exclusive or co-occurring set of genes were investigated using pair-wise Fisher’s Exact test. Moreover*,* comparison of oncogenic pathway alteration frequencies among *MGMT*-methylated and not-methylated cases was performed, by considering that a case was altered in a given pathway if one or more pathway genes had a pathogenic alteration, as previously described^22^.

Supervised analysis of mutational signatures was performed using *YAPSA^23^*. The package provides different sets of mutational signatures, including the COSMIC (Catalogue of Somatic Mutations in Cancer) and PCAWG (Pan-Cancer Analysis of Whole Genomes) SNV signatures and the PCAWG Indel signatures; both SNV and indels mutational signatures were assessed. In detail, 17 Indel signatures were tested, based on a classification of Indels into 83 features, and Doublet Base Signatures (DBS) based on somatic di-nucleotide exchanges.

Finally, homologous recombination deficiency (HRD) was evaluated via the CHORD^24^ algorithm, and results were compared with mutational signatures analysis. Differential representation of mutational signatures and HRD cases between *MGMT*-methylated and not-methylated cases was assessed.

1. Zhang Z, Hernandez K, Savage J, et al: Uniform genomic data analysis in the NCI Genomic Data Commons. Nat Commun 12:1226, 2021

2. Zhang J, Bajari R, Andric D, et al: The International Cancer Genome Consortium Data Portal. Nat Biotechnol 37:367-369, 2019

3. Cerami E, Gao J, Dogrusoz U, et al: The cBio cancer genomics portal: an open platform for exploring multidimensional cancer genomics data. Cancer Discov 2:401-4, 2012

4. Cancer Genome Atlas Research Network. Electronic address aadhe, Cancer Genome Atlas Research N: Integrated Genomic Characterization of Pancreatic Ductal Adenocarcinoma. Cancer Cell 32:185-203 e13, 2017

5. Cao L, Huang C, Cui Zhou D, et al: Proteogenomic characterization of pancreatic ductal adenocarcinoma. Cell 184:5031-5052 e26, 2021

6. Bailey P, Chang DK, Nones K, et al: Genomic analyses identify molecular subtypes of pancreatic cancer. Nature 531:47-52, 2016

7. Colaprico A, Silva TC, Olsen C, et al: TCGAbiolinks: an R/Bioconductor package for integrative analysis of TCGA data. Nucleic Acids Res 44:e71, 2016

8. Bady P, Delorenzi M, Hegi ME: Sensitivity Analysis of the MGMT-STP27 Model and Impact of Genetic and Epigenetic Context to Predict the MGMT Methylation Status in Gliomas and Other Tumors. J Mol Diagn 18:350-361, 2016

9. Love MI, Huber W, Anders S: Moderated estimation of fold change and dispersion for RNA-seq data with DESeq2. Genome Biol 15:550, 2014

10. Leek JT, Johnson WE, Parker HS, et al: The sva package for removing batch effects and other unwanted variation in high-throughput experiments. Bioinformatics 28:882-3, 2012

11. Yu G, Wang LG, Han Y, et al: clusterProfiler: an R package for comparing biological themes among gene clusters. OMICS 16:284-7, 2012

12. Gu Z, Hubschmann D: Simplify enrichment: A bioconductor package for clustering and visualizing functional enrichment results. Genomics Proteomics Bioinformatics, 2022

13. Badia IMP, Velez Santiago J, Braunger J, et al: decoupleR: ensemble of computational methods to infer biological activities from omics data. Bioinform Adv 2:vbac016, 2022

14. Schubert M, Klinger B, Klunemann M, et al: Perturbation-response genes reveal signaling footprints in cancer gene expression. Nat Commun 9:20, 2018

15. Moffitt RA, Marayati R, Flate EL, et al: Virtual microdissection identifies distinct tumor- and stroma-specific subtypes of pancreatic ductal adenocarcinoma. Nat Genet 47:1168-78, 2015

16. Collisson EA, Sadanandam A, Olson P, et al: Subtypes of pancreatic ductal adenocarcinoma and their differing responses to therapy. Nat Med 17:500-3, 2011

17. Gu Z, Schlesner M, Hubschmann D: cola: an R/Bioconductor package for consensus partitioning through a general framework. Nucleic Acids Res 49:e15, 2021

18. Yoshihara K, Shahmoradgoli M, Martinez E, et al: Inferring tumour purity and stromal and immune cell admixture from expression data. Nat Commun 4:2612, 2013

19. Aran D, Hu Z, Butte AJ: xCell: digitally portraying the tissue cellular heterogeneity landscape. Genome Biol 18:220, 2017

20. Li B, Severson E, Pignon JC, et al: Comprehensive analyses of tumor immunity: implications for cancer immunotherapy. Genome Biol 17:174, 2016

21. Mayakonda A, Lin DC, Assenov Y, et al: Maftools: efficient and comprehensive analysis of somatic variants in cancer. Genome Res 28:1747-1756, 2018

22. Sanchez-Vega F, Mina M, Armenia J, et al: Oncogenic Signaling Pathways in The Cancer Genome Atlas. Cell 173:321-337 e10, 2018

23. Hubschmann D, Jopp-Saile L, Andresen C, et al: Analysis of mutational signatures with yet another package for signature analysis. Genes Chromosomes Cancer 60:314-331, 2021

24. Nguyen L, J WMM, Van Hoeck A, et al: Pan-cancer landscape of homologous recombination deficiency. Nat Commun 11:5584, 2020
